# CXCL10/SLC11A1 Axis Exacerbates Septic Liver Injury by Regulating Neutrophil Extracellular Traps Formation to Drive Macrophage Pro‑Inflammatory Polarization

**DOI:** 10.1002/advs.76424

**Published:** 2026-07-13

**Authors:** Haiping Lin, Shicong Zheng, Pei Song, Jie Chang, Zewei Chen, Cang Li, Min Yu, Lude Wang, Shian Yu

**Affiliations:** ^1^ Department of General Surgery Jinhua Central Hospital Teaching Hospital of Mathematical Medicine College Zhejiang Normal University Zhejiang China; ^2^ Central Laboratory and Precision Medicine Center Affiliated Jinhua Hospital Zhejiang University School of Medicine Zhejiang China; ^3^ Key Laboratory of Nutrition and Metabolism Research for Oncology Affiliated Jinhua Hospital Zhejiang University School of Medicine Zhejiang China

**Keywords:** CXCL10, macrophage polarization, neutrophil extracellular trap, septic liver injury, SLC11A1

## Abstract

Intra‐abdominal infection frequently progresses to sepsis, where the liver is an early and commonly injured organ. In a cecal ligation and puncture (CLP) mouse model combined with bulk and single‑cell RNA sequencings, we observed marked neutrophil infiltration in the liver that correlated with injury severity. Sepsis‑associated neutrophils displayed a pro‑inflammatory phenotype and specifically upregulated the divalent metal transporter solute carrier family 11 member 1 (SLC11A1). Conditional knockout of *Slc11a1* in neutrophils (*Ly6G*‐Cre^+^ *Slc11a1* ^f/f^) significantly alleviated liver injury and improved survival. Mechanistically, SLC11A1 drives intracellular Fe^2^^+^ accumulation and reactive oxygen species production via the Fenton reaction, promoting the formation of neutrophil extracellular traps (NETs). Hepatocytes were found to secrete C‐X‐C motif chemokine ligand 10 (CXCL10) through nuclear factor kappa B (NF‑κB) activation, which both recruit neutrophils and stimulates the JAK/STAT1/SLC11A1 axis, thereby enhancing NETs‑mediated pro‑inflammatory macrophage polarization. Clinically, peripheral blood CXCL10 levels correlated with liver injury markers in sepsis patients. Neutralization of CXCL10 using an anti‐CXCL10 antibody or liver‐specific knockdown of CXCL10 via Adeno‐Associated Virus (AAV) reduced NETs formation and attenuated liver damage in CLP mice. This study delineates a “CXCL10–SLC11A1–NETs” signaling axis that exacerbates sepsis‑induced liver injury, offering a novel target for therapeutic intervention.

## Graphical abstract

In sepsis‐induced liver injury, marked neutrophil infiltration correlates with disease severity. Neutrophil SLC11A1 drives intracellular Fe^2^^+^ and ROS via the Fenton reaction, promoting NETs formation. Hepatocyte‐derived CXCL10 recruits neutrophils and activates the JAK/STAT1/SLC11A1 axis, enhancing NETs‐mediated inflammation. Targeting the CXCL10–SLC11A1–NETs axis alleviates liver damage, offering a promising therapeutic strategy.

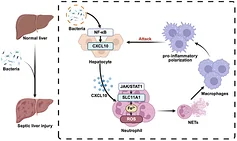

## 1 Introduction

Intra‐abdominal infection is a common complication following abdominal surgeries, such as enterectomy and hepatectomy [1, 2]. These infections can progress to sepsis, a severe systemic inflammatory response with a mortality rate of 15–20% [3]. If uncontrolled, sepsis further culminates in multiple organ dysfunction, at which point the mortality rate exceeds 30% [4, 5, 6]. Due to its extensive vascular network, the liver is among the earliest and most frequently affected organs during sepsis, particularly in cases stemming from intra‐abdominal foci [7, 8].

Cytokines serve as pivotal regulators in the pathogenesis and progression of sepsis‐induced liver dysfunction [7, 9]. As central mediators of the immune response, pro‐inflammatory cytokines—such as tumor necrosis factor‐α (TNF‐α), interleukin‐1β (IL‐1β), and interleukin‐6 (IL‐6)—are rapidly released following septic insults to orchestrate the hepatic inflammatory cascade. However, uncontrolled or excessive cytokine release is detrimental. This hyperactivation of the cytokine network drives hepatocellular injury, cholestasis, mitochondrial impairment, and microcirculatory disturbances.

C‐X‐C motif chemokine ligand 10 (CXCL10) is a pro‐inflammatory chemokine belonging to the CXC chemokine family [10]. It plays key roles in recruiting C‐X‐C chemokine receptor type 3^+^ (CXCR3^+^) immune cells and regulating inflammatory responses. CXCL10 has been implicated in various forms of organ injury. For instance, in acute lung injury, vascular endothelial cells upregulate CXCL10, which disrupts endothelial junction integrity and thereby exacerbates pulmonary inflammation and vascular leakage [11]. In the liver, sinusoidal endothelial cells recruit hepatic iNKT cells and CD4^+^ T cells via CXCL10, contributing to drug‐induced liver injury [12]. However, whether CXCL10 influences the severity of liver injury or serves as a reliable indicator of hepatic dysfunction in sepsis‐induced liver damage remains unclear.

SLC11A1 is a divalent metal ion antiporter, primarily responsible for transporting ferrous iron (Fe^2^^+^) and manganese (Mn^2^^+^), and is predominantly expressed on the phagosomal membranes of macrophages and neutrophils [13]. By exporting essential metal ions such as iron and manganese from the phagosome, SLC11A1 restricts pathogen survival and replication [14]. In addition, alterations in the intraphagosomal ionic environment—particularly changes in iron levels—can modulate NADPH oxidase activity, thereby regulating reactive oxygen species (ROS) production and enhancing microbial killing capacity [15]. However, recent studies have suggested that excessive expression of SLC11A1 may contribute to tissue injury [16, 17]. In breast cancer, neoadjuvant chemotherapy has been reported to induce elevated SLC11A1 expression in neutrophils, which promotes increased formation of neutrophil extracellular traps (NETs) and subsequently leads to vascular endothelial damage [16]. Consistently, SLC11A1 is also found to be upregulated in circulating immune cells in sepsis models. Previous studies have suggested that this upregulation may contribute to pathogen clearance by immune cells; however, whether such increased expression simultaneously mediates liver injury during sepsis remains unclear.

In this study, we used a cecal ligation and puncture (CLP) model that recapitulates sepsis‐induced liver injury resulting from intra‐abdominal infection to characterize the progression of hepatic damage during sepsis. Our findings reveal that hepatocytes secrete CXCL10 to recruit neutrophils and, through upregulation of *Slc11a1* expression within these neutrophils, disrupt their intracellular redox homeostasis. This disruption shifts the hepatic immune microenvironment toward a pro‐inflammatory state, thereby exacerbating sepsis‐associated liver injury. More importantly, in septic patients, peripheral blood CXCL10 levels correlate with the severity of liver injury. Furthermore, neutralizing CXCL10 in septic animal models significantly attenuates the extent of hepatic damage.

## 2 Materials and Methods

### 2.1 Animal Care and CLP/Sham Model

This study utilized 8‐ to 12‐week‐old male C57BL/6J mice obtained from GemPharmatech Co., Ltd. (Jiangsu, China). The animals were housed under controlled environmental conditions, with ambient temperature maintained at 20–26°C, relative humidity at 40–60%, and a 12‐hour light/dark cycle. Food and water were provided ad libitum. All animal care procedures and experimental protocols were conducted in accordance with animal research ethics regulations and were approved by the Experimental Animal Welfare and Ethics Committee of Jinhua Hospital, affiliated with Zhejiang University School of Medicine (Approval No. AL‐JHYY2025199).

Mice were fasted for 12 h prior to CLP surgery. Anesthesia was administered under sterile conditions by inhalation of 2% isoflurane mixed with 1 L/min oxygen. A 1.5 cm midline abdominal incision was made to access the peritoneal cavity. The cecum was ligated at approximately two‐thirds of the distance from its base to the distal end, and a single through‐and‐through puncture was made in the distal cecum using an 18‐gauge needle to induce sepsis. Before returning the cecum to the abdominal cavity, a small amount of fecal content was gently extruded from the puncture site. The abdominal incision was closed in layers using 5‐0 silk suture. Postoperatively, fluid resuscitation was performed by subcutaneous injection of 1 mL of 0.9% sterile saline [18]. Sham‐operated mice underwent identical surgical procedures, including laparotomy, but without cecal ligation or puncture.

For intra‐portal vein injection of Anti‑Ly6G (#1A8, Bio X Cell, USA), the abdominal cavity was opened, and the intestines were gently displaced to the left using sterile cotton swabs. The pancreas was then reflected to expose the portal vein, which lies at its base. A needle was inserted into the portal vein approximately 2 mm below the pancreas, and the injection was performed using either isotype control antibody or Anti‑Ly6G at a dose of 10 mg/kg body weight. After injection, a dry sterile cotton swab was used to apply pressure to the puncture site. The needle was slowly withdrawn while the swab was rotated to seal the injection site and prevent blood reflux. Pressure was maintained on the portal vein for 30 seconds, followed by repositioning of the intestines and closure of the abdominal cavity.

### 2.2 Hematoxylin and Eosin (H&E) Staining

Liver tissues were collected from Sham and CLP mice 24 h post‐surgery. Residual blood was cleared by perfusion with normal saline via the inferior vena cava, followed by fixation in 4% paraformaldehyde for 24 h. The fixed tissues were then dehydrated, embedded in paraffin, and sectioned at a thickness of 3 µm. Sections were deparaffinized in xylene and rehydrated through a graded ethanol series. Next, the sections were stained with hematoxylin for 5 min, rinsed under running tap water, differentiated in 1% acid alcohol for 3–10 seconds, and counterstained with eosin for 30 seconds. Finally, the stained sections were dehydrated through an ascending ethanol series, cleared in xylene, and mounted with neutral resin. The severity of liver injury was comprehensively evaluated based on alterations in hepatocyte volume, nuclear morphology, structural integrity of the hepatic cords, and the degree of immune cell infiltration.

### 2.3 Peripheral Blood Analysis

Blood samples were collected at 24 h post‐surgery. After centrifugation at 3000–4000 × g for 10 min, the serum was separated. Serum levels of alanine aminotransferase (ALT) and aspartate aminotransferase (AST) were measured using commercial assay kits (#ALT01 and #AST01, respectively; Ningbo Purui Biotechnology Co., Ltd., China) according to the manufacturer's instructions. Absorbance readings were performed on a microplate reader, and enzyme concentrations were calculated based on standard curves.

Peripheral blood was collected via retro‑orbital puncture at 24 h post‐surgery into ethylenediaminetetraaceticacid (EDTA)‑coated tubes. Neutrophil counts (both percentage and absolute number) were determined using a TECOM automatic hematology analyzer according to the manufacturer's instructions. Values were reported as neutrophils per microliter of blood and as a percentage of total white blood cells.

### 2.4 RNA Sequencing (RNA‐Seq) and Bioinformatics Analysis

Total RNA was extracted from tissue samples using TRIzol reagent (Thermo Fisher Scientific, USA), and RNA integrity was assessed with an Agilent 2200 TapeStation system. Only samples with an RNA integrity number (RIN) ≥7.0 were included in subsequent procedures. cDNA libraries were constructed with the TruSeq Stranded mRNA Library Prep Kit (Illumina, Inc.) following the manufacturer's protocol. Briefly, poly(A)+ mRNA was enriched from 1 µg total RNA using oligo(dT) magnetic beads and fragmented to 200–600 bp via divalent cation–mediated cleavage at 85°C for 6 min. First‐strand and second‐strand cDNA was synthesized using the fragmented mRNA as a template. Strand specificity was preserved by incorporating dUTP during second‐strand synthesis, followed by enzymatic degradation of the dUTP‐containing strand using uracil DNA glycosylase. The resulting cDNA fragments were subjected to end repair, A‐tailing, and adapter ligation. The first‐strand cDNA was then purified and amplified by PCR to generate the final sequencing libraries. Library quality was verified on an Agilent 2200 TapeStation system, followed by paired‐end 150 bp sequencing on a DNBSEQ‐T7 platform (MGI Tech Co., Ltd., China), producing approximately 20 million reads per sample.

Raw sequencing reads were processed for quality‐controlled with FastQC, and adapter sequences along with low‐quality bases were trimmed using Trim Galore. Clean reads were aligned to the reference genome (GRCh38) using STAR under default parameters. Gene expression was quantified with featureCounts, and differential expression analysis was performed using DESeq2. Principal component analysis (PCA) was conducted to evaluate intra‐group consistency and inter‐group variation. Functional enrichment analysis of differentially expressed genes was carried out through the Gene Ontology (GO) and Kyoto Encyclopedia of Genes and Genomes (KEGG) database via clusterProfiler and Gene Set Enrichment Analysis (GSEA).

### 2.5 Tissue Collection and Single Cell Preparation

Liver tissues were collected from mice at 24 h post‐surgery. After perfusion via the inferior vena cava to clear residual blood, the liver was perfused with DMEM containing 1 mg/mL collagenase IV. The liver was then excised and minced into small fragments. The tissue fragments were further digested in a solution of 1 mg/mL collagenase IV and 0.1 mg/mL DNase I at 37°C for 30 min under constant stirring at 800 g. The resulting suspension was filtered through a 70 µm cell strainer, and the filtrate was centrifuged and resuspended to obtain a single‑cell suspension of hepatic origin.

For hepatic immune cell isolation, after filtration and washing, the cells were resuspended in 5 mL of 40% Percoll solution. The cell suspension was carefully layered on top of a 70% Percoll gradient using a low‑speed pipetting. Centrifugation was performed at 800 × g for 20 min with the brake off. The upper layer (containing dead cells, epithelial cells, and fat) was discarded, and the interphase containing immune cells was collected into a new tube. The collected fraction was diluted with DMEM to a total volume of 15 mL, mixed by gentle inversion, and centrifuged at 600 × g for 7 min at 4°C. The supernatant was then discarded, and the resulting cell pellet represented the isolated immune cells from liver tissues [19].

For the isolation of neutrophils from liver tissue, the immune cell suspension obtained above was subjected to positive selection magnetic sorting (#480058, Biolegend, USA). Cells were adjusted to 1×10^8^ cells/mL in ice‐cold MojoSort Buffer (#480017, Biolegend, USA). For each 10^7^ cells, labeling was performed with 10 µL Biotin‐Antibody Cocktail (15 min on ice). After washing, cells were incubated with 10 µL Streptavidin Nanobeads (15 min on ice). Following an additional wash, cells were resuspended in at least 500 µL of buffer for magnetic separation.

Bone marrow‐derived neutrophils (BMDNs) and bone marrow‐derived macrophages (BMDMs) were isolated from the femurs and tibias of C57BL/6J mice using a density gradient centrifugation method. Briefly, bone marrow cells were flushed from dissected bones with ice‐cold phosphate‐buffered saline (PBS) containing 2% fetal bovine serum (FBS). For BMDMs isolation, after red blood cell lysis, cell suspensions were prepared and cultured in complete medium supplemented with 20 ng/mL macrophage colony‑stimulating factor (M‑CSF) to allow differentiation into BMDMs. For BMDNs isolation, after red blood cell lysis, cell suspensions were layered onto a three‐layer Percoll gradient (52%, 64%, and 72% in PBS). Following centrifugation at 1,200 × g for 30 min at room temperature without brake, neutrophils were collected from the interface between the 64% and 72% layers. Cells were then washed, counted, and resuspended in RPMI‐1640 medium supplemented with 10% FBS for subsequent experiments. The purity of the isolated neutrophils, assessed by flow cytometry exceeded 90%, and cell viability, determined by trypan blue exclusion, consistently exceeded 95%.

### 2.6 Immunohistochemistry (IHC) Staining

Paraffin‐embedded tissue sections (3 µm thick) were deparaffinized in xylene and rehydrated through a graded ethanol series. Antigen retrieval was performed by heating the sections in citrate buffer (pH 6.0) at 95°C for 20 min. Endogenous peroxidase activity was blocked by incubating with 3% hydrogen peroxide for 10 min at room temperature. The sections were then incubated overnight at 4°C with primary antibodies (Table S2), followed by incubation with horseradish peroxidase (HRP)‐conjugated secondary antibodies for 1 hour at room temperature. Immunoreactive signals were visualized using 3,3'‐diaminobenzidine (DAB) substrate, and the sections were counterstained with hematoxylin. Images were acquired using a bright‐field microscope.

### 2.7 Flow Cytometry for Immune Profiling

A single‑cell suspension was prepared as described above. To block nonspecific Fc‑mediated binding, cells were incubated with anti‑mouse CD16/CD32 antibody on ice for 10 min. For immunophenotyping, cells were then stained on ice for 30 min with the following antibody panels: Panel 1—CD45 (PE‑CY7), CD11b (FITC), F4/80 (PE), CD86 (PerCP‑Cy5.5), CD206 (APC); Panel 2—CD45 (PE‑CY7), CD11b (FITC), F4/80 (PE), Ly6C (PerCP‑Cy5.5), MHC II (BV421), Ly6G (APC). After staining, cells were washed with fluorescence‐activated cell sorting buffer (PBS containing 2 % FBS and 2 mM EDTA). Viability was assessed using the Zombie NIR Fixable Viability Kit (#423106, Biolegend, USA). All antibodies used in the flow cytometry are listed in Table S2. Flow cytometry was conducted on a BD FACSCanto II (BD, USA), and data analysis was performed using FlowJo v10.8 (Waters, USA).

### 2.8 Protein Extraction and Immunoblotting

For protein extraction, cell lysates were prepared from isolated tissue cells or cultured cell lines using RIPA buffer supplemented with protease and phosphatase inhibitors. Immunoblotting was performed according to established procedures, and target protein signals were visualized using an enhanced chemiluminescence (ECL) detection system. β‑Actin was used as a loading control. Detailed information on the antibodies used is provided in Table S2.

### 2.9 Single‐Cell RNA Sequencing (scRNA‐Seq) and Bioinformatics Analysis

Following preparation of single‐cell suspensions, the cell suspension was loaded into a 10x Genomics Chromium microfluidic chip compatible with the 3' v3.1/v4 chemistry. Using the Chromium X system, each cell was labeled with a unique barcode. Subsequent steps, including reverse transcription of barcoded RNA into cDNA and construction of sequencing libraries (involving cDNA amplification, end repair, adapter ligation, and size selection), were performed strictly according to the manufacturer's protocol for the 10x Genomics Chromium Single Cell 3' Kit (10x Genomics, Inc., USA).

For data processing, raw sequencing data were initially quality‐controlled and mapped using Cell Ranger to generate a gene expression matrix. This matrix was then imported into Scanpy for downstream analysis. The analysis pipeline included quality filtering (retaining cells with at least 100 expressed genes and removing doublets via sc.pp.scrublet), log‐normalization, and batch effect correction using Harmony. Cells were clustered via a graph‐based Louvain algorithm, and cell types were annotated by correlating gene expression patterns with known marker genes.

Differential expression analysis between clusters was performed using the Wilcoxon rank‐sum test. The biological significance of marker genes was interpreted through GO pathway enrichment analyses using gseapy.gsva. Pseudotime analysis was conducted using Scanpy's diffusion pseudotime algorithm to reconstruct cellular progression trajectories. A neighbor graph was built from highly variable genes, a root cell was defined, and pseudotime values were calculated to order cells along developmental or transitional paths. RNA velocity analysis was conducted by quantifying spliced and unspliced transcripts with scVelo, followed by modeling transcriptional dynamics using the stochastic model in Scanpy. Velocity vectors were projected onto UMAP embeddings to infer the direction and timing of cell‐state transitions.

### 2.10 RNA Extraction and Quantitative Real‐Time PCR (RT‐qPCR)

Total RNA was extracted using the EZBioscience RNA Extraction Kit (#EZB‐RN4, EZBioscience, USA) according to the manufacturer's protocol. cDNA was synthesized from 1 µg of total RNA using a reverse transcription kit following the manufacturer's instructions. RT‑qPCR was performed on a QuantStudio Dx instrument (Thermo Fisher Scientific, USA) with SYBR Green Master Mix (#Q431, Vazyme, China), with each sample analyzed in triplicate technical replicates. Gene expression levels were quantified using the 2−ΔΔCt method, with ACTB or Actb serving as the endogenous control. All experiments were conducted with three independent biological replicates. The primer sequences, designed and synthesized by Sangon Biotech (Shanghai, China), are provided in Table S1.

### 2.11 Immunofluorescence Staining

Immunofluorescence co‑localization of Ly6G and SLC11A1 was performed on mouse liver sections. Following antigen retrieval and blocking, sections were incubated with primary antibodies against Ly6G and SLC11A1, followed by Alexa Fluor 488‑ and 594‑conjugated secondary antibodies, respectively. Nuclei were stained with 4',6‐diamidino‐2‐phenylindole (DAPI). Images were acquired by confocal microscopy, and co‑localization was quantified using Manders’ overlap coefficients calculated in ImageJ (NIH, USA).

The spatial relationship between NETs and M1‐ or M2‑type macrophages was visualized by immunofluorescence co‑staining for myeloperoxidase (MPO) and CD86 or CD206. After antigen retrieval and blocking, tissue sections were incubated with primary antibodies against MPO and either CD86 or CD206, followed by Alexa Fluor 488‑ and 594‑conjugated secondary antibodies, respectively. Nuclei were counterstained with DAPI. Images were acquired by confocal microscopy, and co‑localization was quantified using Manders’ overlap coefficients calculated in ImageJ (NIH, USA).

NETs were visualized in cultured cells and tissue sections using immunofluorescence staining for citrullinated histone H3 (Cit‐H3) and MPO. For cell‐based assays, neutrophils seeded on coverslips were stimulated with CXCL10 (100 ng/mL) for 24 h, fixed in 4% paraformaldehyde, permeabilized with 0.1% Triton X‐100, and blocked with 5% bovine serum albumin. For tissue sections, formalin‐fixed, paraffin‐embedded or frozen sections were deparaffinized, subjected to antigen retrieval, and similarly blocked. All samples were incubated overnight at 4°C with primary antibodies against Cit‐H3 and MPO, followed by appropriate fluorescent secondary antibodies (Alexa Fluor 594 for Cit‐H3 and Alexa Fluor 488 for MPO) and nuclear counterstaining with DAPI. Coverslips or slides were mounted with antifade medium and imaged using a fluorescence microscope. In cell‐based assays, NETs were quantified as the percentage of neutrophils undergoing NETosis, defined as MPO^+^/Cit‐H3^+^ cells with a nuclear size exceeding twice the mean area of unstimulated control nuclei. In tissue sections, NETs were quantified as the percentage of the field area occupied by Cit‐H3^+^/MPO^+^ extracellular structures.

All antibodies used in the Immunofluorescence staining are listed in Table S2.

### 2.12 Intracellular reactivate oxygen species (ROS) and Fe^2+^ Measurement

Intracellular ROS levels in neutrophils were measured by incubating cells with 20 µM DCFDA/H_2_DCFDA probe (#ab113851, Abcam, UK) at 37°C for 30 min. After washing, the cells were subjected to flow cytometry analysis using the FITC channel (485/535 nm). ROS levels were quantified as the mean fluorescence intensity (MFI) of DCFDA/H_2_DCFDA‑FITC.

Intracellular Fe^2+^ levels in neutrophils were measured by incubating cells with 1 µM FerroOrange probe (#HY‐D1913, MedChemExpress, USA) at room temperature for 30 min. After washing, the cells were subjected to flow cytometry analysis using the PE channel (540/585 nm). Fe^2+^levels were quantified as the MFI of FerroOrange‐PE.

### 2.13 Cell Line and Primary Cell Culture

The AML‐12 and HEK293T cell lines were obtained from the Cell Bank of the Chinese Academy of Sciences (Shanghai, China) and were maintained in DMEM supplemented with 10% FBS. BMDNs or BMDMs isolated from mice were cultured in RPMI‑1640 medium containing 10% FBS. Primary hepatocytes, renal tubular epithelial cells, and alveolar epithelial cells were cultured in commercial proprietary media (#iCell‑d017, #iCell‑u001, #iCell‑a005, Cellverse Co., Ltd., China) specifically formulated for each respective primary cell type. All cells were incubated at 37°C in a humidified atmosphere with 5% CO_2_.

Primary hepatocytes, alveolar epithelial cells, and renal tubular epithelial cells were first pre‐stimulated with Lipopolysaccharide (LPS) (1 µg/mL) for 8 h to induce an inflammatory state. These activated parenchymal cells were then co‐cultured directly with freshly isolated BMDNs in a co‐culture chamber (#3420, Corning, USA) for 16 h. After co‐culture, BMDNs were collected for RNA extraction. Parallel monocultures of BMDNs served as experimental controls.

### 2.14 Cytokine Array

Cytokine expression was assessed using a commercial mouse cytokine array kit (#ARY006, R&D Systems, USA) according to the manufacturer's protocol. Membranes were blocked and then incubated overnight at 4°C with conditioned medium (CM) samples. The CM was collected from primary hepatocytes isolated from the livers of CLP‐ and Sham‐operated mice after 16 h of in vitro culture. Prior to membrane incubation, each sample was pre‐mixed with a biotinylated detection antibody cocktail. Following the overnight incubation, membranes were probed with streptavidin–HRP conjugate for signal detection. Image analysis was performed to measure spot pixel density, background signals were subtracted, and duplicate spots were averaged to determine relative cytokine levels across samples.

### 2.15 Enzyme‐linked Immunosorbent Assay (ELISA)

ELISA assays were performed using commercial assay kits (#EK168, #EK268, Multi Sciences, China) according to the manufacturer's protocol. Briefly, 50  µL of each sample or standard and 50  µL of detector antibody cocktail were added to the designated wells, mix gently, and the plate was incubated at room temperature for 1  hour. Following incubation, the wells were thoroughly washed. TMB development solution was then added, and the plate was incubated for 10 min. Finally, stop solution was added, and the absorbance was measured at 450 nm using a microplate reader.

### 2.16 Dual‐Luciferase Reporter Assay

To investigate the direct functional interaction between Rela and the *Cxcl10* promoter, dual‐luciferase reporter assays were performed using the Dual‑Luciferase Reporter Assay System (#DL101, Vazyme, China) according to the manufacturer's protocol. The putative promoter region of the mouse *Cxcl10* gene was cloned into the pGL3‑basic luciferase reporter vector. Truncated or site‑directed mutant variants of the *Cxcl10* promoter were generated to assess the contributions of specific Rela‑binding sites.

AML‑12 cells were seeded into 12‑well plates at a density of approximately 4 × 10^4^ cells per well and cultured overnight at 37°C under 5% CO_2_ until reaching approximately 60% confluency. For transfection, 1 µg of the *Cxcl10* promoter reporter plasmid (wild‑type, truncated, or mutant) together with 100  ng of the Renilla luciferase internal control plasmid (pRL vector) were co‑transfected into shCtrl or shRela AML‑12 cells using Lipofectamine 3000 transfection reagent (#L3000015, ThermoFisher Scientific, China). After 6 h, the medium was replaced with fresh complete medium containing 10% FBS. 24 h post‑transfection, cells were stimulated with either 1 µg/mL LPS (#HY‐D1056, MedChemExpress, USA) or 100  ng/mL phorbol 12‐myristate 13‐acetate (PMA) (#HY‐18739, MedChemExpress, USA). Cell lysis was performed 48  h after transfection. Firefly and Renilla luciferase activities were sequentially measured using a luminometer. To normalize for transfection efficiency, firefly luciferase activity was calibrated against Renilla luciferase activity.

### 2.17 Chromatin Immunoprecipitation (ChIP) assay

ChIP assay was performed using the SimpleChIP Enzymatic Chromatin IP Kit (#9003, Cell Signaling Technology, USA) according to the manufacturer's protocol. Cells were fixed with formaldehyde, lysed, and the chromatin was fragmented by a combination of Micrococcal Nuclease digestion and sonication to yield fragments corresponding to 1–5 nucleosomes. Chromatin extracts containing DNA fragments were subjected to immunoprecipitation with an anti‐Rela antibody (#8242, Cell Signaling Technology, USA) or isotype IgG antibody. The immunoprecipitated DNA was then washed, purified, and analyzed by RT‐qPCR. The primer sequences used for ChIP‑qPCR are provided in Table S1.

### 2.18 Neutrophil Chemotaxis Assay

Neutrophils were isolated from mouse bone marrow using the density gradient centrifugation described above. Then, 1×10^5^ cells (in 200 µL) were seeded into the upper chamber of a 24‑well Transwell insert (#3421, Corning, USA). Both the upper and lower chambers contained serum‑supplemented medium. Graded concentrations of CXCL10 (0, 20, and 100 ng/mL) were added to the lower chamber. After 4 h of incubation, the number of transmigrating neutrophils was quantified. The cells were fixed with 4% paraformaldehyde for 30 min, stained with crystal violet for 30 min, and then gently washed to remove excess dye. The transmigrated neutrophils were observed under a microscope and counted using ImageJ software.

### 2.19 Adeno‐Associated Virus (AAV) Transfection

AAV8 (serotype 8) vectors used to knock down *Cxcl10* were custom‑packaged by Obio Technology Corp., Ltd. (Shanghai, China) using the pAAV‑TBG‑Luc2‑mir30shRNA‑WPRE (target sequence: GATGTCTGAATCCGGAATCTA). *Cxcl10* shRNA (AAV8‑Cxcl10‑shRNA) or control shRNA (AAV8‑NC‑shRNA) was delivered via tail vein injection at a dose of 5 × 10^1^ ^1^ viral genomes (v.g.) per mouse. Four weeks post‑injection, fluorescence distribution was assessed using an in vivo bioluminescence imaging (IVIS) system. Knockdown efficiency was evaluated by RT‑qPCR and ELISA, after which subsequent experiments were performed.

### 2.20 Clinical Sample Collection

On the day of intensive care unit (ICU) admission, 20 mL of peripheral blood was collected from patients with sepsis secondary to intra‐abdominal infection. For patients unable to provide consent personally, informed consent was obtained from their immediate family members. This study was approved by the Ethics Committee of Jinhua Hospital, affiliated with Zhejiang University School of Medicine. Written informed consent was obtained from all participants prior to sample collection ([Research] 2021‐Ethics Review‐319).

### 2.21 Statistical Analysis

Continuous variables were compared between two groups using the Student's t‐test (for normally distributed data) or the Wilcoxon rank‑sum test (for non‑normally distributed data). Comparisons among multiple groups were performed with one‑way analysis of variance (ANOVA) or the Kruskal–Wallis test, as appropriate. Categorical variables were analyzed using the chi‑square test or Fisher's exact test. Overall survival was estimated by the Kaplan–Meier method, and differences between survival curves were evaluated with the log‑rank test. All statistical analyses were carried out with GraphPad Prism v9.0 (GraphPad Software, La Jolla, CA, USA) and R v4.4.2 (CRAN, https://www.cran.r‑project.org). Unless otherwise noted, a two‑sided P‑value < 0.05 was regarded as statistically significant.

## 3 Results

### 3.1 Increased Neutrophil Infiltration in the Liver Exacerbates Injury Severity Following CLP

To simulate clinical cases of sepsis‐induced liver injury in patients with abdominal infections, we established a murine model of sepsis via CLP to recapitulate the pathological features of liver injury (Figure 1A). Histopathological assessment of liver tissues (H&E staining) revealed hepatic damage in CLP‐operated mice compared to Sham controls, as evidenced by pyknotic or absent nuclei, diminished hepatocyte volume, dissociation from liver cord structures, and increased inflammatory cell infiltration (Figure 1B). In line with these histopathological findings, elevated serum levels of ALT and AST provided biochemical confirmation of liver injury (Figure 1C).

**FIGURE 1:**
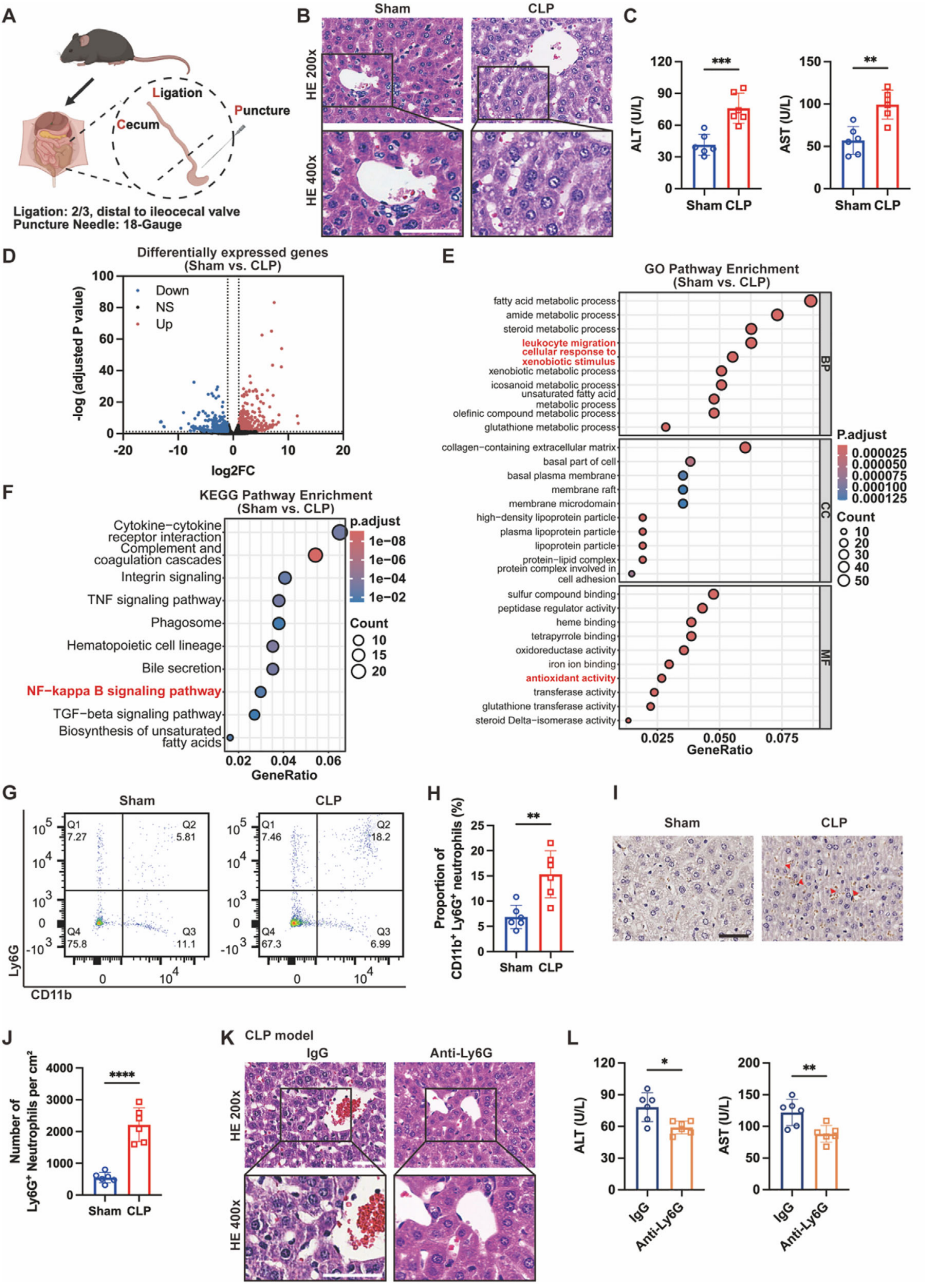
Increased Neutrophil Infiltration in the Liver Exacerbates Injury Severity Following CLP. (A) Schematic illustration of the cecal ligation and puncture (CLP) model used to induce sepsis in mice. (B) Representative hematoxylin and eosin (H&E) staining of liver injury in the CLP and Sham groups 24 h post‑surgery. Scale bar = 50 µm. (C) Quantification of serum ALT and AST levels in the CLP and Sham groups 24 h post‑surgery. (D) RNA‐Seq analysis of liver tissues from CLP and Sham groups; a volcano plot shows differentially expressed genes (DEGs) between the two groups. (E,F) GO (E) and KEGG (F) enrichment analyses of DEGs identified by RNA‐Seq analysis of liver tissues. (G, H) Flow cytometric analysis (G) and quantification (H) of the proportion of neutrophils within the CD45^+^ cells in the livers of CLP and Sham groups 24 h post‑surgery, with neutrophils identified as CD11b^+^ Ly6G^+^ cells. (I,J) Representative immunohistochemical staining of Ly6G (red arrows) in liver tissues from Sham and CLP groups 24 h post‑surgery (I), with quantification of Ly6G^+^ neutrophils per cm^2^ (J). Scale bar = 50 µm. (K) Representative H&E staining of liver injury in CLP mice treated with either isotype control antibody or anti‐Ly6G antibody (10 mg/kg) via the portal vein, assessed 24 h post‑surgery. Scale bar = 50 µm. (L) Quantification of serum ALT and AST levels in CLP mice treated with either isotype control antibody or anti‐Ly6G antibody (10 mg/kg), assessed 24 h post‑surgery. In (C), (H), (J), (L) data represent mean ± SD; unpaired two‐tailed Student's t test. *P < 0.05, **P < 0.01, ***P < 0.001, ****P < 0.0001 and ns *P* > 0.05 between the indicated groups.

To characterize the transcriptional alterations in the liver following CLP‐induced injury, we performed RNA sequencing on liver tissues from both CLP and Sham‐operated mice, with PCA confirming the distinct expression profiles between the two groups (Figure S1A). Differential gene expression analysis revealed a marked upregulation of numerous neutrophil‐associated genes—including specific neutrophil markers (e.g. *S100a9* and *Ngp*) and key neutrophil chemokines (e.g. *Ccl6* and *Cxcl1*)—in the CLP group (Figure 1D). Subsequent GO and KEGG analysis further revealed a significant enrichment of pathways involved in leukocyte migration and oxidative stress (Figure 1E,F). Additionally, we observed a significant enrichment of the NF‐κB pathway, a master regulator of inflammation and immunity. Consistently, GSEA demonstrated an upregulation of this pathway in the CLP group (Figure S1B), which was further confirmed by elevated expression of p‐Rela and p‐IκBα (Figure S1C). Similarly, treatment of the normal mouse hepatocyte cell line AML‐12 with LPS, a pathogen‐associated molecular pattern (PAMP), also resulted in activation of the NF‐κB pathway (Figure S1D). Together, these findings demonstrate enhanced inflammatory activation—particularly involving neutrophils—during the progression of septic liver injury.

Previous studies have demonstrated that although neutrophils employ multiple mechanisms to eliminate pathogens and damaged cells, these same mechanisms can also inflict damage on hepatocytes [20]. We confirmed a significant increase in hepatic neutrophil infiltration in CLP‐operated mice compared to Sham controls using flow cytometry and immunohistochemistry (Figure 1G–J). To investigate the impact of neutrophil accumulation on the severity of CLP‐induced liver injury, we administered an anti‐Ly6G antibody via the portal vein. This route was selected to achieve a localized depletion of hepatic neutrophils [21], as the portal vein delivers blood directly to the liver, without significantly reducing neutrophil levels in the peripheral blood (Figure S1E–H). Following hepatic neutrophil depletion, we observed a marked attenuation of liver injury, as evidenced by only variably sized cytoplasmic vacuoles in hepatocytes without significant nuclear alterations, along with reduced serum levels of ALT and AST (Figure 1K,L). These results demonstrate that the increased accumulation of neutrophils in the liver contributes to the exacerbation of liver injury during sepsis.

### 3.2 Hepatic Neutrophils Exhibit Pro‐Inflammatory Features in CLP Model

To further characterize liver‐infiltrating neutrophils in the CLP model, we conducted a single‐cell RNA sequencing (scRNA‐seq) on liver tissues from CLP‐operated and Sham‐operated mice (Figure 2A). Based on marker gene expression, cells were annotated as hepatocytes, erythroid‐like and erythroid precursor cells (EPCs), liver sinusoidal endothelial cells (LSECs), Kupffer cells, monocyte‐derived macrophages (MDMs), neutrophils, T cells, B cells, NK cells, and dendritic cells (DCs) (Figure S2A,B).

**FIGURE 2:**
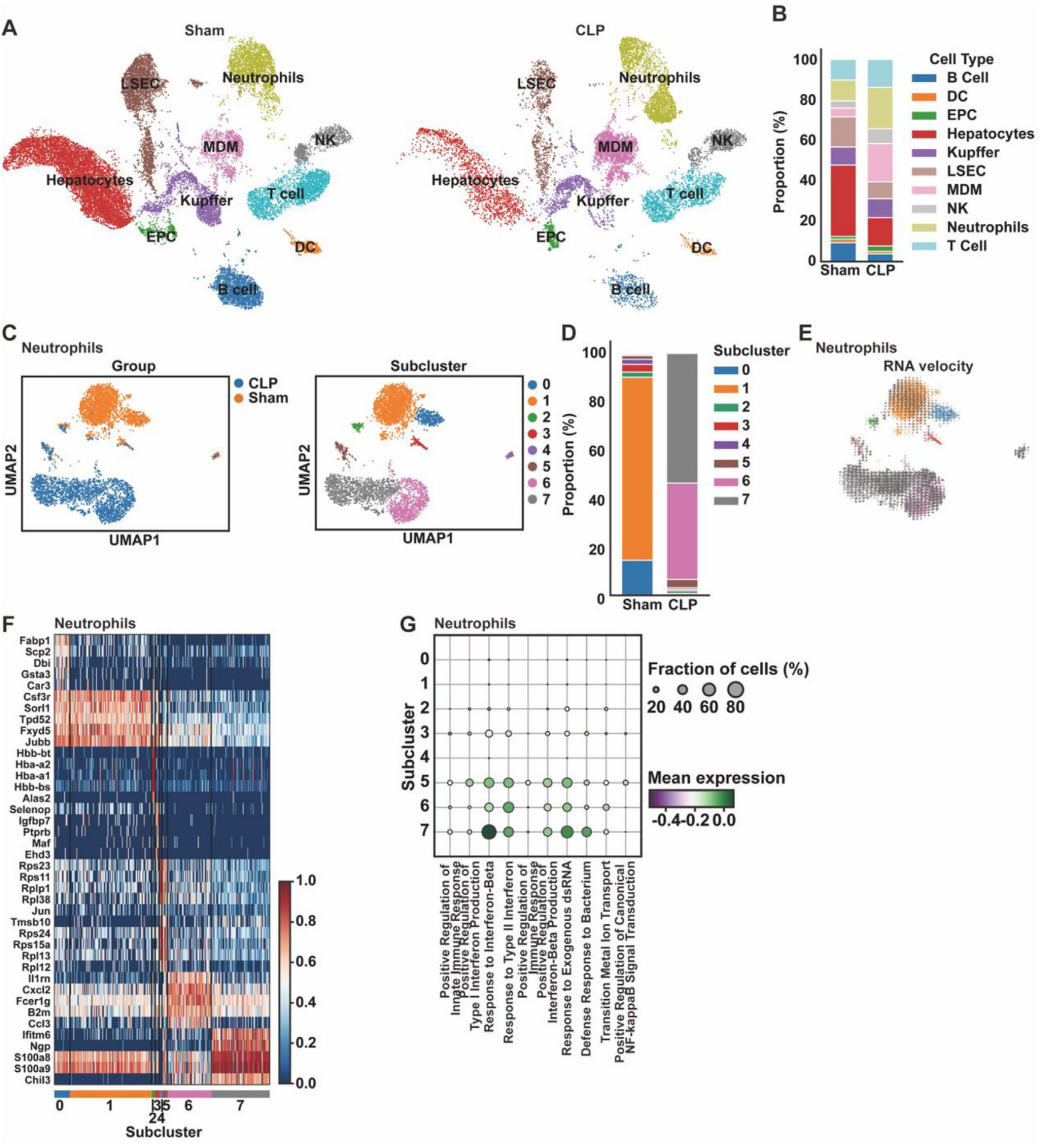
Hepatic Neutrophils Exhibit Pro‐Inflammatory Features in CLP Model. (A) Uniform manifold approximation and projection (UMAP) visualization of liver scRNA‐seq data from the CLP and Sham groups. Cells were annotated as hepatocytes, erythroid‐like and erythroid precursor cells (EPCs), liver sinusoidal endothelial cells (LSECs), Kupffer cells, monocyte‐derived macrophages (MDMs), neutrophils, T cells, B cells, NK cells, and dendritic cells (DCs). (B) The proportions of distinct cell types in the CLP and Sham groups. (C) UMAP of neutrophil from the CLP and Sham groups. Neutrophil were divided into 7 distinct cell subclusters through unsupervised clustering. (D) The proportions of distinct neutrophil subclusters in the CLP and Sham groups. (E) Streamline plot of RNA velocity vectors projected onto the UMAP embedding, colored by neutrophil subclusters. Arrows indicate the inferred direction and magnitude of transcriptional changes for individual cells. (F) Heatmap showing the highly expressed genes within each of the 7 distinct neutrophil subclusters. (G) Dot plot of GSVA scores showing the enrichment of significant Gene Ontology (GO) pathways (top 10 ranked by adjusted p‑value) across different neutrophil subclusters.

Our analysis revealed a significant increase in the proportions of both neutrophils and MDMs in CLP‐operated mice compared to Sham controls (Figure 2B). Further examination of neutrophils showed distinct transcriptional profiles between the two groups (Figure 2C,D). RNA velocity and pseudotime trajectory analyses collectively indicated that the majority of sepsis‐associated neutrophils at the late stages of the differentiation trajectory, consistent with previously reported mature neutrophil characteristics at sites of infection (Figure 2E and Figure S2C). Analysis of differentially expressed genes among neutrophil subclusters identified Clusters 3, 4, 5 and 6—predominantly composed of CLP neutrophils—as exhibiting a pro‐inflammatory phenotype. This phenotype was characterized by the upregulation of genes involved in neutrophil recruitment and promotion of inflammatory cytokine expression (Figure 2F). Further Gene Set Variation Analysis (GSVA) of Gene Ontology (GO) terms revealed that Clusters 3, 4, 5, and 6 were enriched not only in immune‐related pathways but also in the ‘Cellular Response to Oxidative Stress’ and the ‘Transition Metal Ion Transport’ pathways (Figure 2G). Collectively, these results suggest that during septic liver injury, infiltrating neutrophils adopt a pro‐inflammatory phenotype, and further implicate metal ion transport‐mediated alterations in oxidative stress within these cells.

We next examined changes in MDMs between the Sham and CLP groups. The MDM subclusters exhibited distinct transcriptional expression profiles between the two groups, with only Cluster 2 maintaining a similar proportion across conditions (Figure S2D,E). Notably, MDMs in the CLP group displayed pro‐inflammatory characteristics. For instance, Cluster 4 showed high expression of complement‐related genes such as *C1qa*, *C1qb*, and *C1qc*, as well as the inflammatory cell recruitment gene *CCR5*; Cluster 5 exhibited elevated expression of pro‐inflammatory genes including *Pycard* and *Fcgr3*; and Cluster 8 demonstrated high expression of the M1 macrophage marker *Saa3* (Figure S2F). Additionally, GSVA enrichment of GO terms revealed significant enrichment of pathways related to inflammatory response and immune cell chemotaxis, particularly in Clusters 4, 5, 6, 7, and 8, which were predominantly associated with the CLP group (Figure S2G). These results suggest that macrophages infiltrating the livers of septic mice are predominantly pro‐inflammatory in phenotype and may play a role in mediating liver injury.

### 3.3 Slc11a1 is Specifically Upregulated in Liver‐Infiltrating Neutrophils and Exacerbates Hepatic Injury in the CLP Model

Beyond the liver, the lungs and kidneys are also frequently damaged during sepsis. To identify key molecules that regulate the pro‐inflammatory state of hepatic neutrophils and thereby exacerbate organ‐specific liver injury in sepsis, we conducted an integrated analysis of single‐cell transcriptomic datasets from Sham and CLP models. These included datasets from the liver (our study), lungs (GSE207651), and kidneys (GSE298053), enabling a comparative investigation across commonly affected organs. Our screening strategy employed the following criteria: (1) specific high expression in neutrophils but not in other immune cells in the liver single‐cell datasets; (2) significant upregulation specifically in the CLP group compared to the Sham group; and (3) absence of neutrophil‐specific high expression in CLP models of kidney or lung injury (Figure 3A and Figure S3A–D). Based on these screening criteria, we identified *Slc11a1* as a significantly and specifically upregulated gene in neutrophils from CLP‐induced livers (Figure 3B,C). Its expression was predominantly localized to neutrophil subcluster 3 in our scRNA‐seq analysis (Figures 3D and 2C).

**FIGURE 3:**
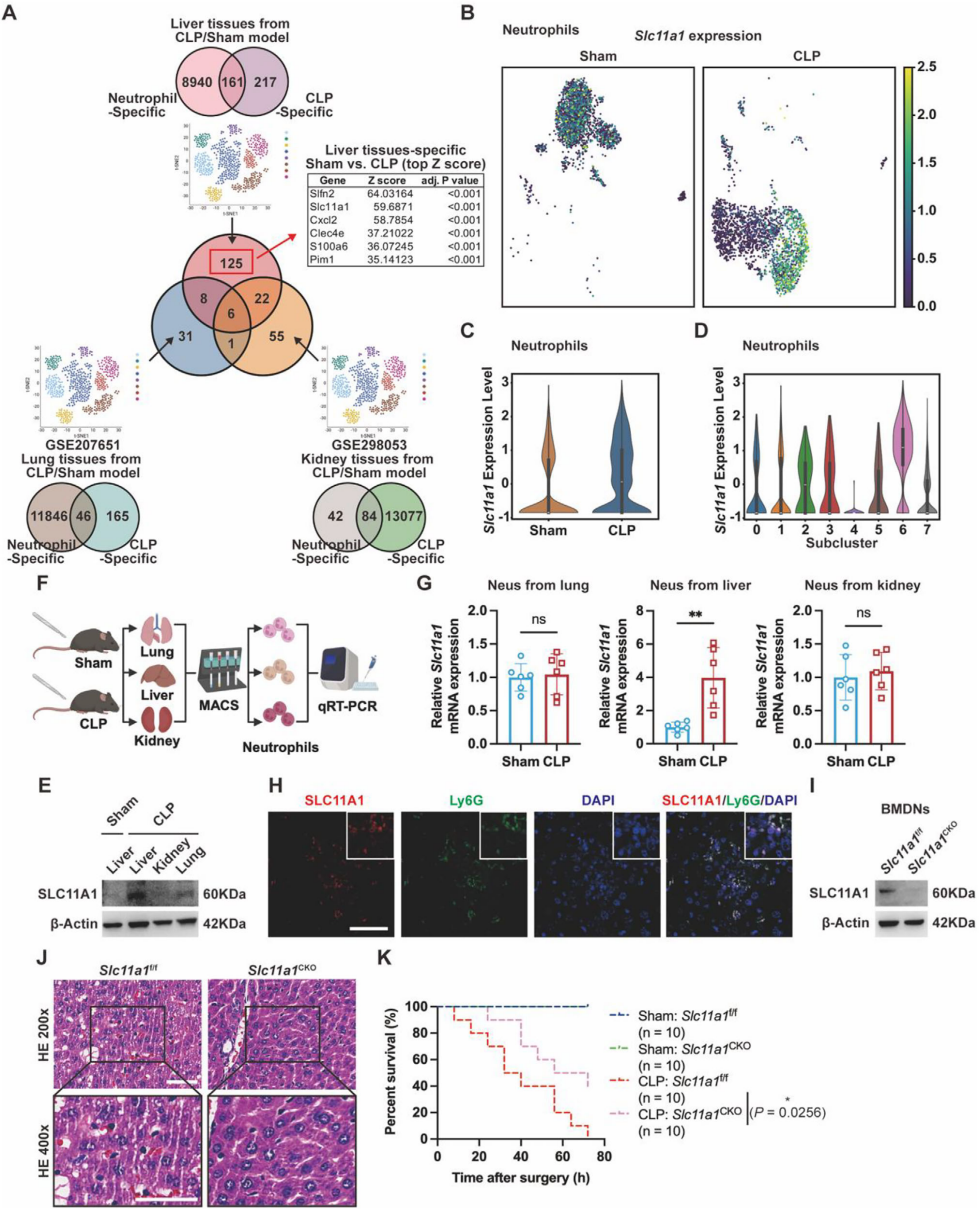
*Slc11a1* is specifically upregulated in liver‐infiltrating neutrophils and exacerbates hepatic injury in the CLP model. (A) Schematic illustration of the screening strategy for identifying genes specifically upregulated in neutrophils within the livers of septic mice, based on integrated single‑cell datasets from the liver, kidney, and lung of CLP and Sham groups. Differentially expressed genes were filtered using an absolute Z score > 30 and adjusted p‐value < 0.01. (B) UMAP of neutrophils for the Sham and CLP groups, featuring *Slc11a1* expression. (C) Expression levels of *Slc11a1* in neutrophils from Sham and CLP groups. (D) Expression levels of *Slc11a1* across distinct neutrophil subclusters. (E) Immunoblotting of SLC11A1 in liver tissues from the Sham group and in liver, kidney and lung tissues from the CLP group, assessed 24 h post‑surgery. (F) Schematic illustration of neutrophil isolation from the lungs, liver, and kidneys of Sham or CLP mice via magnetically activated cell sorting, followed by RNA extraction for RT‐qPCR analysis. (G) *Slc11a1* mRNA levels in lung‐, liver‐, and kidney‐derived neutrophils from Sham and CLP groups 24 h post‑surgery, determined by RT‐qPCR. (H) Immunofluorescence co‐staining of SLC11A1 and Ly6G in the liver of CLP‐operated mice, assessed 24 h post‑surgery. Scale bar = 50 µm. (I) Immunoblotting of SLC11A1 in bone marrow‐derived neutrophils isolated from *Slc11a1* ^CKO^ and *Slc11a1* ^f/f^ mice. (J) Representative H&E staining images of liver injury in *Slc11a1* ^CKO^ mice compared with *Slc11a1* ^f/f^ mice in the Sham and CLP model, assessed 24 h post‑surgery. (K) Kaplan–Meier survival curves showing the percentage survival of *Slc11a1* ^CKO^ and *Slc11a1* ^f/f^ mice subjected to Sham or CLP over 72 h. In (C), (G) data represent mean ± SD; unpaired two‐tailed Student's t test. In (K) log‐rank test. *P < 0.05, **P < 0.01, ****P < 0.0001 and ns *P* > 0.05 between the indicated groups.

To validate our single‐cell analysis findings, we collected peripheral blood, liver, kidney, and lung tissues from CLP models, along with liver tissues from Sham models. Immunoblotting analysis revealed specific upregulation of SLC11A1 protein in the livers of CLP mice (Figure 3E). Consistently, examination of sorted neutrophils from these tissues confirmed the specific high expression of *Slc11a1* exclusively in hepatic neutrophils from CLP models (Figure 3F,G). Furthermore, fluorescence staining of liver sections from CLP and Sham groups demonstrated strong SLC11A1 expression in CLP hepatic neutrophils (Figure 3H). Together, these data validate the specific upregulation of *Slc11a1* in liver‐infiltrating neutrophils in the CLP model and imply its functional contribution to aggravated neutrophil‐driven liver injury.

To determine whether neutrophil‐specific SLC11A1 expression contributes to CLP‐induced liver injury, we generated mice with conditional knockout (CKO) of *Slc11a1* in neutrophils (*Slc11a1* ^CKO^) (Figure 3I and Figure S3E,F). Compared to control mice (*Slc11a1* ^f/f^), *Slc11a1* ^CKO^ mice exhibited attenuated liver injury, as evidenced by preserved hepatic architecture with minimal nuclear pyknosis or karyolysis, intact liver cord organization, and significantly lower serum ALT and AST levels (Figure 3J and Figure S3G). Notably, conditional knockdown of *Slc11a1* in neutrophils in the CLP model conferred a survival benefit (Figure 3K). These results confirm that elevated *Slc11a1* expression in hepatic neutrophils promotes the exacerbation of sepsis‐induced liver injury.

### 3.4 Slc11a1 Aggravates Sepsis‐Induced Liver Injury via ROS‐Mediated NETs Formation in Neutrophils

We next investigated the mechanism by which neutrophil‐specific upregulation of *Slc11a1* exacerbates CLP‐induced liver injury. As a transporter mediating lysosomal iron transport, SLC11A1 facilitates cytosolic Fe^2^^+^ accumulation under septic conditions. Given that excess Fe^2^^+^ can catalyze hydroxyl radical (•OH) generation from H_2_O_2_ via the Fenton reaction, we hypothesized that SLC11A1 enhances reactive oxygen species (ROS) production by increasing intracellular Fe^2^^+^ levels (Figure 4A). Using FerroOrange and DCFH‐DA probes to assess Fe^2^^+^ and ROS levels, respectively, in sorted hepatic neutrophils, we observed higher Fe^2^^+^ and ROS in CLP group than in Sham controls (Figure 4B,C). Furthermore, under septic conditions, *Slc11a1* ^CKO^ mice exhibited reduced Fe^2^^+^ and ROS levels in hepatic neutrophils compared to *Slc11a1* ^f^/^f^ controls (Figure 4D,E). Moreover, treatment of bone marrow‐derived neutrophils (BMDNs) isolated from WT C57BL/6J mice with ferric ammonium citrate (FAC), a membrane‐permeable iron source, significantly increased intracellular ROS levels, and this elevation was reversed by deferoxamine (DFO) (Figure 4F). These results confirm that *Slc11a1* augments ROS production by elevating intracellular Fe^2^^+^ in neutrophils.

**FIGURE 4:**
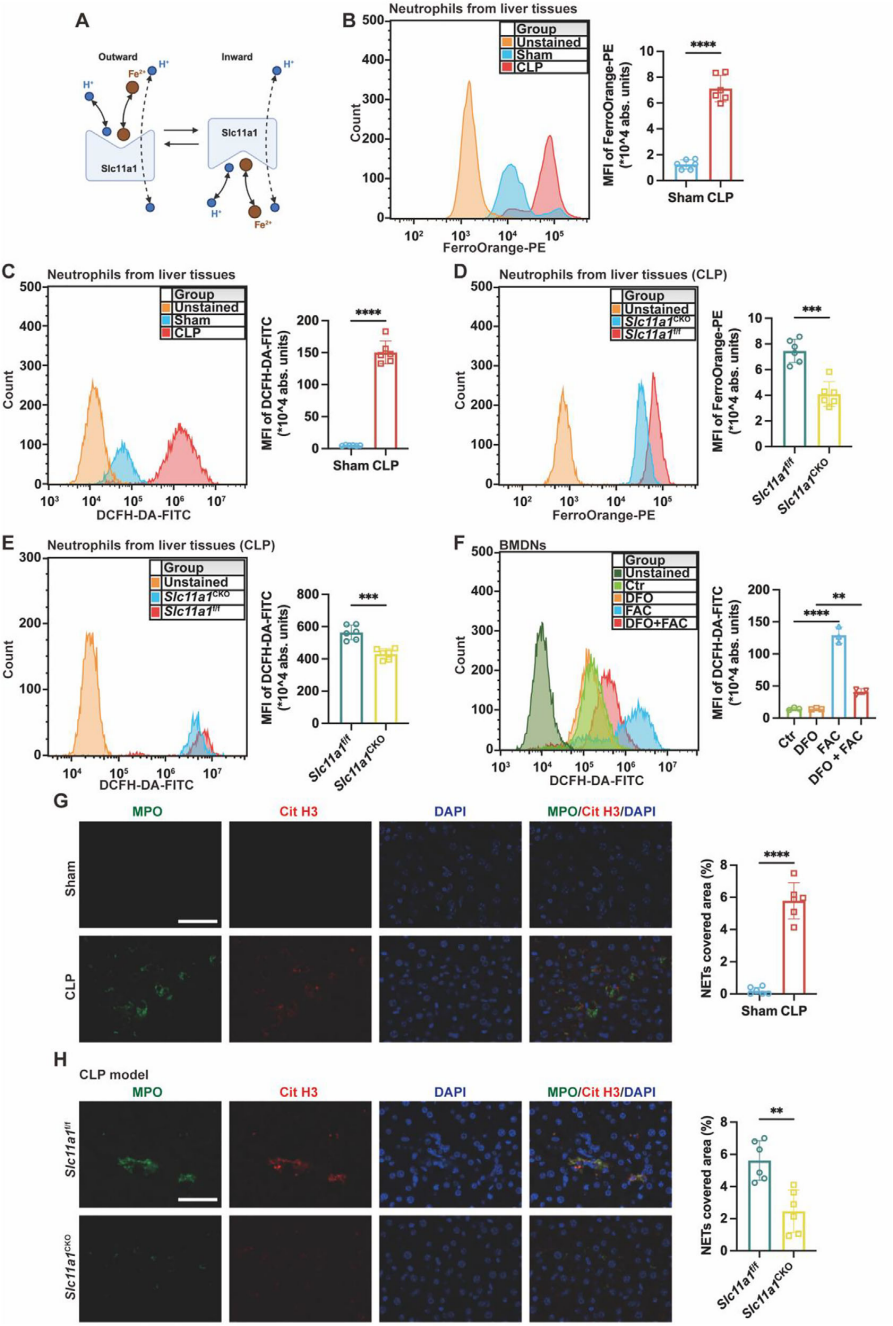
*Slc11a1* Aggravates Sepsis‐Induced Liver Injury via ROS‐Mediated NETs Formation in Neutrophils. (A) Schematic illustration of the SLC11A1 transporter mediating transition metal ion uptake coupled with proton symport through an alternating access mechanism, concurrent with an uncoupled proton leak pathway. (B) Intracellular Fe^2^^+^ levels in neutrophils isolated from the livers of Sham and CLP groups 24 h post‑surgery, measured by flow cytometry using the FerroOrange probe. (C) Intracellular ROS levels in neutrophils isolated from the livers of Sham and CLP groups 24 h post‑surgery, measured by flow cytometry using the DCFH‐DA probe. (D) Intracellular Fe^2^^+^ levels in neutrophils isolated from the livers of CLP‐treated *Slc11a1* ^f/f^ and *Slc11a1* ^CKO^ mice, assessed 24 h post‑surgery, measured by flow cytometry using the FerroOrange probe. (E) Intracellular ROS levels in neutrophils isolated from the livers of CLP‐treated *Slc11a1* ^f/f^ and *Slc11a1* ^CKO^ mice, assessed 24 h post‑surgery, measured by flow cytometry using the DCFH‐DA probe. (F) Intracellular ROS levels in BMDNs treated with deferoxamine (DFO) (10 µM) or ammonium iron citrate (FAC) (100 µM) for 24 h, measured by flow cytometry using the DCFH‐DA probe. (G) Neutrophil extracellular traps (NETs) formation assessed in liver tissues from Sham and CLP groups by immunofluorescence co‐staining of myeloperoxidase (MPO) and citrullinated histone H3 (Cit‐H3), assessed 24 h post‑surgery. Scale bar = 50 µm. (H) NETs formation assessed in liver tissues from CLP‐treated *Slc11a1* ^f/f^ and *Slc11a1* ^CKO^ mice by immunofluorescence co‐staining of MPO and Cit‐H3, assessed 24 h post‑surgery. Scale bar = 50 µm. In (B), (C), (D), (E), (G), (H) data represent mean ± SD; unpaired two‐tailed Student's t test. In (F) data represent mean ± SD; one‐way ANOVA with multiple comparisons test. **P < 0.01, ***P < 0.001, ****P < 0.0001 and ns *P* > 0.05 between the indicated groups.

Increased ROS levels in neutrophils serve as a key marker of neutrophil activation following the recognition of pathogens or inflammatory signals [22, 23]. On the one hand, elevated ROS can oxidize the proteins, lipids, and DNA of pathogens to lead to their death; on the other hand, increased ROS acts as a critical trigger for the neutrophil extracellular traps (NETs) formation [24]. Notably, NETs have been implicated in various tissue and organ injuries, suggesting their potential role in sepsis‐induced liver damage. Accordingly, we first examined NETs formation in the livers of the Sham and CLP groups. The results revealed a significant increase in NETs in the CLP group compared to the Sham group (Figure 4G). Additionally, NETs content in the livers of *Slc11a1* ^CKO^ mice of the CLP model was significantly reduced compared to that in *Slc11a1* ^f^/^f^ mice (Figure 4H). Next, following NETs removal using DNase I, we observed reduced severity of sepsis‐induced liver injury, as indicated by the restoration of normal hepatocyte nuclear morphology, the disappearance of cytoplasmic loosening, and the absence of nuclear separation (Figure S4A). Moreover, DNase I treatment significantly reduced serum ALT and AST levels (Figure S4B). These results suggest that sepsis‐induced specific high expression of *Slc11a1* in liver neutrophils regulates the intracellular Fe^2+^ distribution to increase ROS production, thereby promoting NETs formation in the liver.

### 3.5 NF‐κB‐Mediated Hepatic CXCL10 Induction Promotes Slc11a1 Expression in Neutrophils via JAK/STAT1 Axis

Given that *Slc11a1* is specifically upregulated in liver‑infiltrating neutrophils—but not in those from the lungs or kidneys—in the CLP model, we hypothesized that hepatocytes may drive this increased expression. To test this hypothesis, we established co‐culture models using mouse primary hepatocytes, alveolar epithelial cells, and renal tubular epithelial cells with BMDNs (Figure 5A). Following pre‐stimulation with LPS to mimic Gram‐negative bacterial infection, we observed significant upregulation of *Slc11a1* in BMDNs co‐cultured with hepatocytes. In contrast, no notable change in *Slc11a1* expression was detected in BMDNs co‐cultured with alveolar epithelial cells or renal tubular epithelial cells (Figure 5B).

**FIGURE 5:**
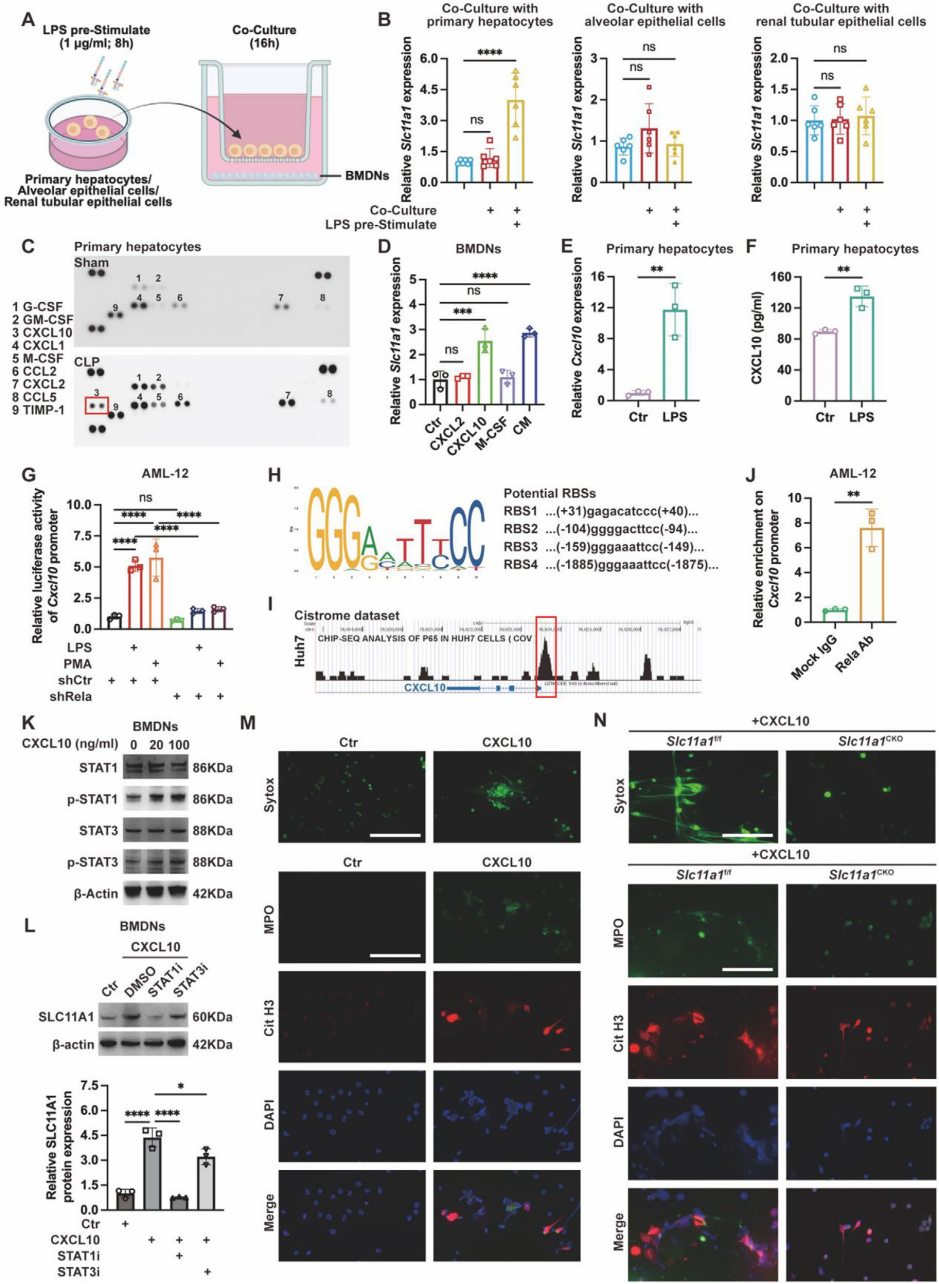
NF‐κB‐Mediated Hepatic CXCL10 Induction Promotes *Slc11a1* Expression in Neutrophils via JAK/STAT1 Axis. (A) Schematic illustration of the co‐culture system with bone marrow‐derived neutrophils (BMDNs) co‑cultured with primary hepatocytes, alveolar epithelial cells, and renal tubular epithelial cells, all pre‐stimulated with LPS. (B) *Slc11a1* mRNA levels in BMDNs co‐cultured with primary hepatocytes, alveolar epithelial cells, or renal tubular epithelial cells for 16 h, determined by RT‐qPCR. (C) Cytokine secretion profiles of primary hepatocytes isolated from the livers of Sham or CLP groups, analyzed using a cytokine array. (D) *Slc11a1* mRNA levels in BMDNs treated with recombinant CXCL2, CXCL10, M‐CSF (100 ng/mL each), conditioned medium (CM) from primary hepatocytes isolated from CLP model mice or vehicle control for 24 h, determined by RT‐qPCR. (E) *Cxcl10* mRNA levels in primary hepatocytes treated with LPS (1 µg/mL) or vehicle control for 24 h, determined by RT‐qPCR. (F) CXCL10 secretion levels in primary hepatocytes treated with LPS (1 µg/mL) or vehicle control for 24 h, determined by ELISA. (G) Relative luciferase activity of the *Cxcl10* promoter in AML‐12 cells transfected with shCtr or shRela and treated with LPS (1 µg/mL) or PMA (100  ng/mL) for 24 h. (H) The JASPAR database predicted 4 potential Rela binding sites (RBSs) within the *Cxcl10* promoter region. (I) Analysis of ChIP‑Seq data from the Cistrome database revealed significant RelA enrichment at the *CXCL10* promoter region in the Huh7 cell line. (J) ChIP‑qPCR validation of RelA binding to the *Cxcl10* promoter in AML‑12 cells. (K) Immunoblot analysis of STAT1 and STAT3 phosphorylation in BMDNs following treatment with recombinant CXCL10 (0, 20, and 100 ng/mL) for 24 h. (L) Immunoblot analysis of SLC11A1 protein expression in BMDNs treated with recombinant CXCL10 (100 ng/mL) in the presence or absence of a STAT1 inhibitor (10 µM) or a STAT3 inhibitor (10 µM) for 24 h. (M) NETs formation assessed in BMDNs treated with recombinant CXCL10 (100 ng/mL) or vehicle control for 24 h, visualized by immunofluorescence staining with Sytox Green or co‐staining for MPO and Cit‐H3. Scale bar = 50 µm. (N) NETs formation assessed in BMDNs from *Slc11a1* ^f/f^ and *Slc11a1* ^CKO^ mice treated with recombinant CXCL10 (100 ng/mL) for 24 h, visualized by immunofluorescence staining with Sytox Green or co‐staining for MPO and Cit‐H3. Scale bar = 50 µm. In (E), (F), (J) data represent mean ± SD; unpaired two‐tailed Student's t test. In (B), (D), (G), (L) data represent mean ± SD; one‐way ANOVA with multiple comparisons test. *P < 0.05, **P < 0.01, ***P < 0.001, ****P < 0.0001 and ns *P* > 0.05 between the indicated groups.

To identify the specific hepatic factor responsible for upregulating *Slc11a1* in neutrophils during sepsis, we isolated primary hepatocytes from the livers of CLP and Sham‐operated mice. These hepatocytes were cultured in vitro for 16 h, after which the conditioned medium (CM) was collected and analyzed for cytokine secretion using a cytokine array. This cytokine array revealed a marked increase in extracellular G‐CSF, GM‐CSF, CXCL10, M‐CSF, CCL2, CXCL2 and CCL5 levels (Figure 5C and Figure S5A). Accordingly, we examined the transcriptional levels of the corresponding cytokines in the RNA‐seq data from the CLP and Sham groups. The results showed that *Cxcl10*, *Csf1*, and *Cxcl2* expressions were all upregulated in the CLP group (Figure S5B). By supplementing BMDNs with CXCL2, M‐CSF, CXCL10, and, as a positive control, CM derived from primary hepatocytes isolated from CLP mice, we found that CXCL10 specifically enhanced *Slc11a1* expression in neutrophils (Figure 5D and Figure S5C). Consistently, LPS stimulation of both mouse primary hepatocytes and the mouse hepatocyte cell line AML‐12 increased both *Cxcl10* transcription and CXCL10 secretion (Figure 5E,F and Figure S5D,E). These findings indicate that hepatocyte‐specific production of CXCL10 during sepsis promotes the upregulation of *Slc11a1* expression in neutrophils.

Next, we investigated the mechanism underlying increased hepatic CXCL10 secretion during sepsis. As described above, the NF‐κB signaling pathway plays a central role in immune responses and inflammatory reactions. Our pathway enrichment analysis of RNA‐seq data from CLP and Sham livers also demonstrated significant enrichment of the NF‐κB pathway (Figure S1C). Based on these findings, we hypothesized that sepsis‐mediated activation of the hepatic NF‐κB signaling pathway drives *Cxcl10* upregulation and CXCL10 secretion. Supporting this hypothesis, stimulation with the NF‐κB agonists PMA robustly increased both *Cxcl10* transcription and CXCL10 secretion in both mouse primary hepatocytes and AML‐12 cells (Figure S5F,G).

In the canonical NF‐κB pathway, the p65 (Rela)/p50 heterodimer primarily translocates to the nucleus to exerts its transcriptional activation function. Using a dual‐luciferase reporter assay, we demonstrated that both LPS and PMA stimulate transcriptional activity at the *Cxcl10* promoter region in AML‐12 cells (Figure 5G). Conversely, Rela knockdown attenuated *Cxcl10* promoter activity (Figure 5G and Figure S5H). To identify the specific binding sites through which the p65/p50 heterodimer regulates the *Cxcl10* promoter region, we performed motif prediction using the JASPAR database, which revealed 4 potential Rela binding sites (RBSs) within the candidate *Cxcl10* promoter region (Figure 5H). To precisely identify the NF‐κB‐responsive elements in the *Cxcl10* promoter, we generated a series of truncated *Cxcl10* promoter fragments. Dual‐luciferase reporter assays revealed that the ‐666 to +100 bp region of the *Cxcl10* promoter is primarily responsible for NF‐κB‐mediated transcriptional regulation (Figure S5I,J). Furthermore, site‐directed mutagenesis of the respective RBSs revealed that mutations in RBS2 and RBS3 significantly reduced NF‐κB‐mediated transcriptional activity, whereas mutations in RBS1 and RBS4 had no significant effect (Figure S5K). Meanwhile, we also searched the Cistrome database and analyzed the ChIP‐Seq results of Rela in human hepatocellular carcinoma Huh7 cells, revealing significant Rela enrichment at the *CXCL10* promoter (Figure 5I). Furthermore, our ChIP‐qPCR analysis confirmed the direct binding of Rela specifically within the promoter region of *Cxcl10* in AML‐12 (Figure 5J). These findings collectively demonstrate that sepsis‐induced activation of hepatic NF‐κB transcriptionally upregulates *Cxcl10* expression and promotes its extracellular secretion.

We next sought to determine how hepatocyte‐derived CXCL10 upregulates *Slc11a1* expression in neutrophils. Previous studies have established that CXCL10 binding to its receptor CXCR3 activates the JAK/STAT pathway, primarily through phosphorylation of STAT1 and STAT3 [25]. Accordingly, we treated BMDNs with recombinant CXCL10 and observed increased phosphorylation of both STAT1 and STAT3 (Figure 5K). However, pharmacological inhibition of STAT1 (Fludarabine, STAT1i)—but not STAT3 (STAT3‐IN‐1, STAT3i)—suppressed *Slc11a1* expression (Figure 5L). These results demonstrate that hepatocyte‐secreted CXCL10 promotes *Slc11a1* expression in neutrophils through STAT1 activation.

To investigate whether CXCL10‑mediated upregulation of *Slc11a1* in neutrophils leads to enhanced NETs formation, we treated BMDNs with CXCL10. The results showed that CXCL10 treatment significantly increased the ability of neutrophils to form NETs (Figure 5M and Figure S5L). Furthermore, the increase in CXCL10‐mediated NETs formation was significantly reduced in *Slc11a1* ^CKO^ mice, confirming that CXCL10 regulates NETs formation through SLC11A1 (Figure 5N and Figure S5M). Taken together, the above results suggest that during sepsis, hepatocytes specifically release CXCL10 into the liver microenvironment, which in turn promotes the upregulation of *Slc11a1* expression in neutrophils via the JAK/STAT1 axis, ultimately facilitating NETs formation.

In addition, given the observed increase in hepatic neutrophil infiltration in the CLP group, we reasoned that CXCL10, a key chemokine, might mediate this recruitment. This hypothesis was confirmed by transwell assays, in which the addition of CXCL10 significantly enhanced neutrophil migration (Figure S5N).

### 3.6 NETs Aggravate Septic Liver Injury by Promoting Pro‐inflammatory Phenotypes in Macrophages

To determine whether increased NETs formation resulting from *Slc11a1* upregulation in neutrophils can directly damage hepatocytes, we isolated NETs from PMA‐stimulated BMDNs and applied them to mouse primary hepatocytes and AML‐12 cells (Figure S6A). Notably, NETs exposure did not significantly inhibit cell viability or induce cell death in either hepatocyte model (Figure S6B,C). This finding suggests that NETs may not contribute to liver injury through direct cytotoxicity toward hepatocytes.

The scRNA‐seq analysis of livers from CLP and Sham models revealed a significant increase in the proportion of macrophages with a pro‐inflammatory phenotype during sepsis (Figure 2D and Figure S2F,G). Given previous studies have reported that NETs regulate monocyte and macrophage activation and differentiation in cancer [26], we hypothesized that NETs may exacerbate liver injury by modulating monocyte and macrophage function. Measurement of macrophage activation markers revealed that hepatic macrophages in the CLP group exhibited elevated CD86 (a marker of classically activated/inflammatory macrophages) and reduced CD206 (a marker of alternatively activated/anti‐inflammatory macrophages) compared to the Sham group (Figure 6A). In addition, we assessed the spatial relationship between NETs and either CD86^+^ or CD206^+^ macrophages in the livers of CLP mice using immunofluorescence staining. The results showed that NETs were spatially proximal to CD86^+^ macrophages but distal from CD206^+^ macrophages (Figure 6B).

**FIGURE 6:**
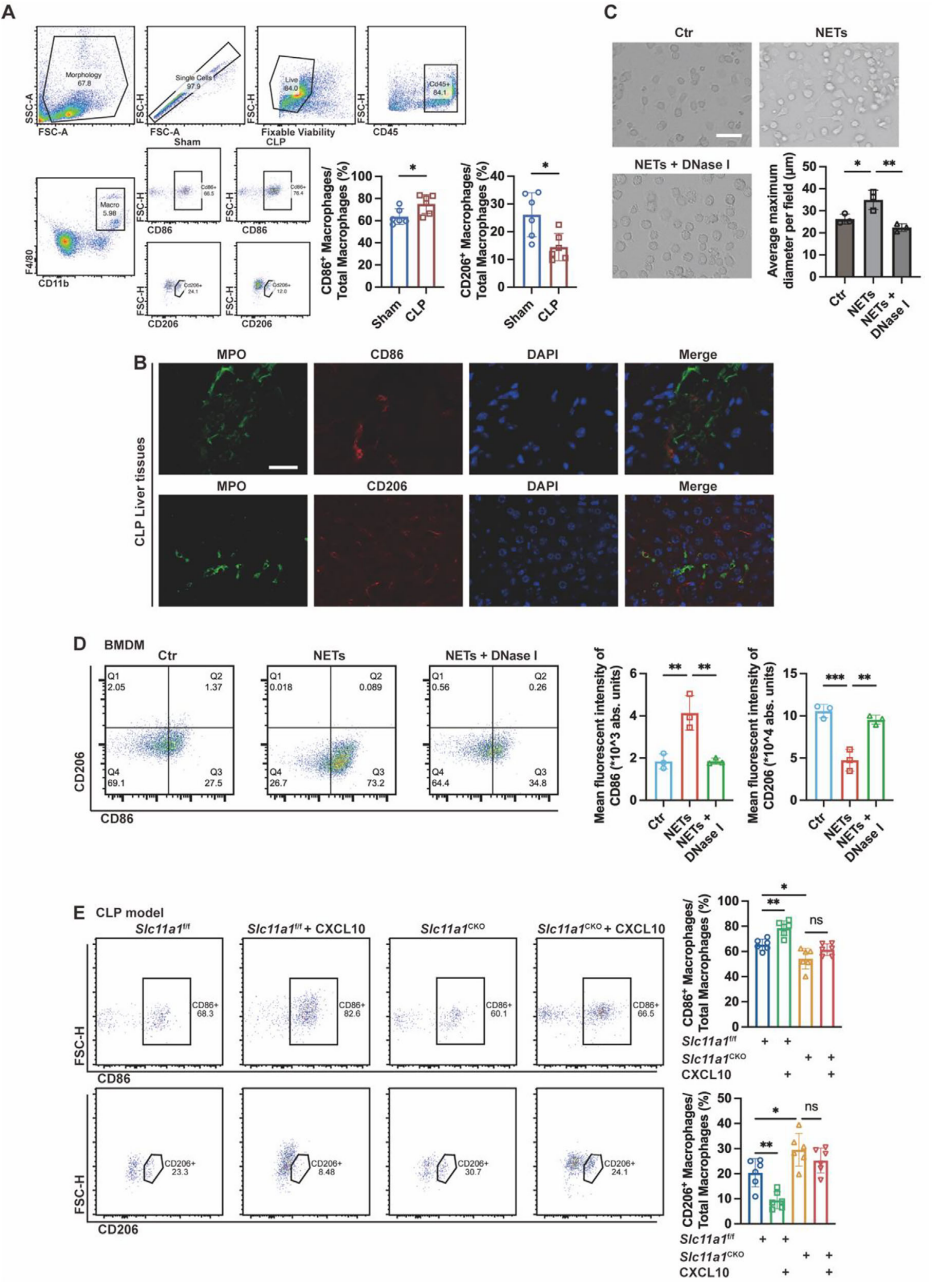
NETs Aggravate Septic Liver Injury by Promoting Pro‐inflammatory Phenotypes in Macrophages. (A) Flow cytometric analysis of CD86^+^ and CD206^+^ macrophage proportions in the Sham and CLP groups 24 h post‑surgery, with the gating strategy shown. (B) Representative immunofluorescence images showing the spatial proximity of MPO (green) with CD86 (red) or CD206 (red) in mice subjected to CLP for 24 h. Scale bar = 50 µm. (C) Representative images showing morphological changes in macrophages treated with or without isolated NETs (2 µg/mL) for 12 h. Scale bar = 50 µm. (D) Flow cytometric analysis of CD86 and CD206 expression levels in BMDMs treated with either NETs (2 µg/mL) or DNase I (20 U/mL) for 12 h. (E) Flow cytometric analysis of CD86^+^ and CD206^+^ macrophage proportions in *Slc11a1* ^CKO^ and *Slc11a1* ^f/f^ mice, with or without CXCL10 (100 ng/mL) treatment, following CLP, assessed 24 h post‑surgery. In (A) data represent mean ± SD; unpaired two‐tailed Student's t test. In (C), (D), (E) data represent mean ± SD; one‐way ANOVA with multiple comparisons test. *P < 0.05, **P < 0.01, ***P < 0.001 and ns *P* > 0.05 between the indicated groups.

To determine whether NETs can drive macrophages toward a pro‐inflammatory phenotype, we treated mouse bone‐marrow–derived macrophages (BMDMs) with NETs. Compared with the control group, NETs‐treated BMDMs exhibited smaller cell bodies, shorter and blunter pseudopodia (Figure 6C), as well as higher CD86 expression and lower CD206 expression (Figure 6D), consistent with an M1‐type polarized state. Furthermore, this shift toward a pro‐inflammatory phenotype was attenuated by DNase I treatment (Figure 6C,D).

To further elucidate how the CXCL10/SLC11A1 axis promotes pro‐inflammatory polarization of macrophages, we administered exogenous CXCL10 to *Slc11a1* ^f^/^f^ and *Slc11a1* ^CKO^ mice subjected to CLP. The CKO mice exhibited reduced pro‐inflammatory activation of hepatic macrophages compared to controls (Figure 6E). Importantly, CXCL10 administration enhanced pro‐inflammatory activation in control mice but showed no significant effect in CKO mice (Figure 6E). These findings demonstrate that CXCL10‐mediated upregulation of *Slc11a1* in neutrophils exacerbates liver injury by enhancing NETs formation, which subsequently promotes the pro‐inflammatory polarization of hepatic macrophages.

Correspondingly, we also examined the inflammatory activation state of monocytes in the liver during sepsis. However, no significant differences in Ly6C and MHC‐II levels were detected between the Sham and CLP groups (Figure S6D). Similarly, the CXCL10/SLC11A1 axis had no significant effect on the inflammatory activation state of monocytes (Figure S6E).

### 3.7 CXCL10 Blockade Attenuates NETs and Ameliorates Septic Liver Injury

To further confirm that hepatocyte‐derived CXCL10 exacerbates liver injury by promoting NETs formation during sepsis, we constructed and packaged AAV8‑CXCL10 shRNA and control AAV8‑NC shRNA viruses (Figure S7A). These were administered via tail vein injection for 4 weeks to achieve conditional knockdown of *Cxcl10*. Luciferase reporter signals accumulated predominantly in the liver, confirming liver‑specific expression (Figure S7B). In primary hepatocytes isolated from the liver, both *Cxcl10* mRNA and CXCL10 secretion levels were significantly decreased (Figure S7C,D). LPS stimulation significantly increased *Cxcl10* mRNA and CXCL10 secretion in the AAV‑NC shRNA group but had no significant effect in the AAV‑*Cxcl10* shRNA group (Figure S7C,D). Furthermore, liver‑specific *Cxcl10* knockdown significantly reduced intrahepatic NETs content following CLP (Figure S7E). It also attenuated the severity of liver injury, as evidenced by preserved hepatic architecture with minimal nuclear pyknosis or karyolysis, intact hepatic cord organization, and lower serum ALT and AST levels (Figure S7F,G).

To validate the correlation between CXCL10 and liver injury in patients with sepsis, we collected peripheral blood from 20 patients who had developed sepsis secondary to abdominal infections in the Intensive Care Unit (ICU) of our center. We examined the correlation between CXCL10 and ALT as well as AST. The results showed that CXCL10 was significantly correlated with both ALT and AST in sepsis patients, suggesting that CXCL10 is associated with the severity of liver injury in these patients (Figure 7A).

**FIGURE 7:**
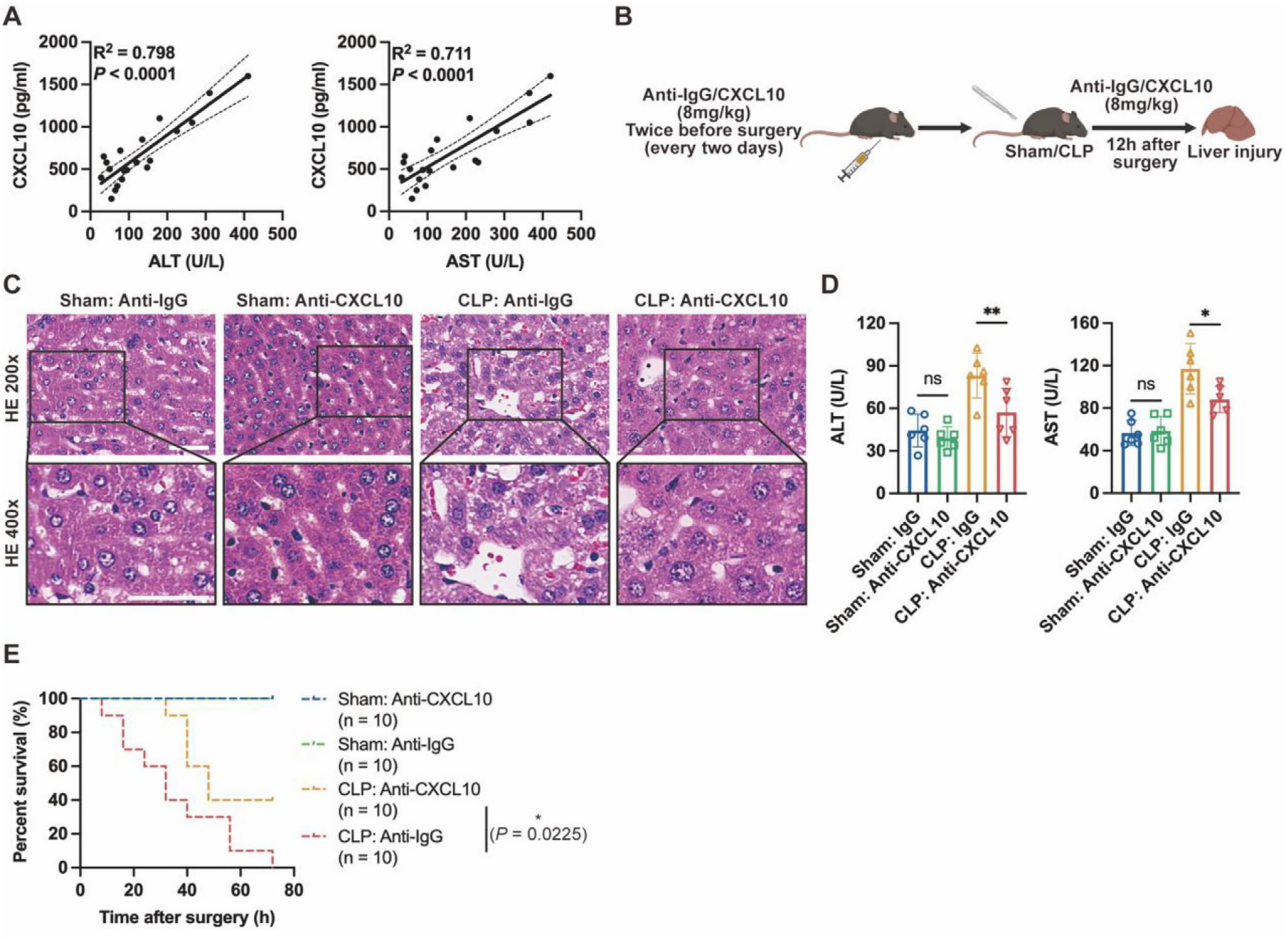
CXCL10 Blockade Attenuates NETs and Ameliorates Septic Liver Injury. (A) Correlation analysis of peripheral blood CXCL10 levels with ALT or AST in patients from JHYY cohort who developed sepsis secondary to abdominal infections. (B) Schematic illustration of the experimental timeline: mice were treated with isotype control antibody or anti‐CXCL10 antibody (8 mg/kg), followed by Sham or CLP operation. An additional dose of isotype control antibody or anti‐CXCL10 antibody (8 mg/kg) was administered 12 h post‐surgery. (C) Representative H&E staining images of liver injury in Sham or CLP groups treated with either isotype control antibody or anti‐CXCL10 antibody, assessed 24 h post‑surgery. Scale bar = 50 µm. (D) Quantification of serum ALT and AST levels in Sham or CLP groups treated with either isotype control antibody or anti‐CXCL10 antibody, assessed 24 h post‑surgery. (E) Kaplan–Meier survival curves showing the percentage survival of mice treated with either isotype control antibody or anti‐CXCL10 antibody and subjected to Sham or CLP over 72 h. In (A) Pearson correlation analysis. In (D) data represent mean ± SD; one‐way ANOVA with multiple comparisons test. In (E) log‐rank test. *P < 0.05, **P < 0.01 and ns *P* > 0.05 between the indicated groups.

We next hypothesized that neutralizing CXCL10 could alleviate septic liver injury. Prior to performing CLP or Sham on mice, we administered two doses of either CXCL10 neutralizing antibody or isotype control antibody at a frequency of once every two days. 12 h after the CLP surgery, we administered another dose of either the CXCL10 neutralizing antibody or isotype control antibody (Figure 7B). The results showed that treatment with the CXCL10 neutralizing antibody reduced the severity of septic liver injury (Figure 7C,D), and improved survival outcomes in the CLP mice (Figure 7E). These findings suggest that neutralizing CXCL10 can alleviate the severity of sepsis‑induced liver injury, which may hold potential for clinical translation.

## 4 Discussion

The liver plays a central and multifaceted role in the pathophysiology of sepsis [7, 9]. As a key immunometabolic organ with a rich blood supply, it contributes to both the early innate immune response and the subsequent regulation of systemic inflammation, acting as a guardian, a modifier, and a target during septic injury [7]. During sepsis, the liver is responsible for clearing pathogens, endotoxins, and inflammatory mediators from the bloodstream [27]. It also synthesizes acute‐phase proteins, regulates glucose and lipid metabolism, and maintains hemostatic balance [7]. However, the liver is also highly susceptible to septic injury. Dysregulated inflammation, impaired microcirculation, and mitochondrial dysfunction can lead to hepatocellular damage, cholestasis, and metabolic derangements [28]. These alterations not only reflect the severity of sepsis but also actively exacerbate systemic inflammation and contribute to the progression of multiple organ dysfunction. Thus, hepatic dysfunction is considered both a marker and a driver of poor outcomes in sepsis [8].

As a unique immune organ, the liver undergoes profound microenvironmental changes during sepsis progression, and these alterations play a critical role in disease development [29, 30]. In this study, based on scRNA‐seq data, we analyzed changes in the microenvironment of the liver, lungs, and kidneys in the CLP‐induced sepsis model. Unlike the marked increase in neutrophil abundance observed in the liver during sepsis, the proportion of neutrophils in the lungs remained relatively stable, and although their proportion increased in the kidneys, the overall number remained low. These findings suggest that neutrophils may play a particularly important regulatory role in sepsis‐induced liver injury.

Consistent with previous reports [20], our in vivo experiments confirmed that the neutrophil infiltration in the liver exacerbates the progression of hepatic injury. Moreover, portal vein administration of a neutrophil‐depleting antibody alleviated liver damage. During infection, neutrophils primarily engage in phagocytosis, degranulation, or the release of NETs through NETosis. Accordingly, we detected an increase in hepatic NETs during sepsis, and the removal of NETs mitigated the severity of liver injury. Notably, although previous studies have reported associations between NETs and liver damage—such as NETs promoting alcohol‐induced or cholestatic liver injury—the role of NETs in sepsis‐induced liver injury has remained underexplored [31, 32]. Here, our study reveals and clarifies this previously overlooked connection.

In further mechanistic investigations, we sought to identify the specific factors that drive neutrophil NETosis and the subsequent NETs release. Based on scRNA‐seq data, we found that *Slc11a1* is specifically upregulated in hepatic neutrophils during sepsis, a finding we subsequently validated experimentally. As a divalent metal transporter located on the membrane of phagosomes or lysosomes, SLC11A1 restricts pathogen access to essential metal ions (mainly Fe^2^^+^) during infection, thereby inhibiting their replication [13]. For example, the host resistance factor SLC11A1 limits the growth of Salmonella and Mycobacterium tuberculosis by depriving them of divalent metal ions [14, 33]. However, in the context of sepsis‐induced liver injury, SLC11A1 in neutrophils not only reduces Fe^2^^+^ within phagosomes but also leads to an accumulation of Fe^2^^+^ in the cytoplasm [13]. This increase in cytoplasmic Fe^2^^+^ fuels a burst of intracellular ROS through the Fenton reaction, which in turn drives NETosis and enhances NETs formation, ultimately aggravating liver injury [34, 35]. Similarly, a recent prospective observational study also suggested that iron metabolism disorders are closely associated with the development of sepsis‑associated liver injury in septic patients [36].

Given that neutrophils and NETs often play a dual role in the overall progression of sepsis—eliminating pathogens and limiting inflammation on one hand, while exacerbating organ injury on the other—simply depleting neutrophils or NETs is not an ideal strategy for managing sepsis‐induced liver damage [37, 38]. Therefore, we considered whether the liver possesses unique mechanisms for recruiting neutrophils or inducing NETs formation, and whether targeting these liver‐specific molecules could offer a more effective strategy for alleviating sepsis‐induced liver injury.

Under harmful stimuli such as sepsis, the liver responds by restructuring the communication network within its microenvironment, utilizing cytokines with either anti‐inflammatory or pro‐inflammatory properties to address corresponding challenges [39, 40, 41, 42]. On one hand, cytokines can directly act on hepatocytes to alleviate damage or recruit other immune cells from the microenvironment to cooperatively protect hepatocytes. For example, fibroblast growth factor 1 (FGF1) alleviates acetaminophen (APAP)‐induced acute liver injury in mice by inhibiting mitochondrial oxidative stress [39]. Additionally, NF‐κB‐dependent IL‐6 mobilizes and recruits myeloid‐derived suppressor cells (MDSCs), thereby controlling the inflammatory response and protecting the liver from inflammatory damage [40]. On the other hand, cytokines secreted by the liver may also contribute to the exacerbation of liver injury. For instance, Kupffer cells produce and release pro‐inflammatory mediators such as tumor necrosis factor‐α (TNF‐α) and interleukin‐1 (IL‐1), triggering an uncontrolled inflammatory response that leads to hepatocyte necrosis and apoptosis, thereby worsening liver damage and potentially progressing to liver failure [41, 42].

In this study, we isolated primary hepatocytes from control and septic liver injury model groups and, after in vitro culture, measured the cytokine levels in the culture supernatants. We found that hepatocytes from the septic model specifically secrete high levels of CXCL10. More importantly, in in vivo models, neutralizing CXCL10 significantly reduced the severity of liver injury and extended the survival of the corresponding mice. In exploring the underlying mechanisms, based on previous reports that CXCL10 binds to its receptor CXCR3 to activate the STAT1 or STAT3 pathways, we examined the activation of these pathways in neutrophils. Although CXCL10 activated both STAT1 and STAT3, it significantly promoted SLC11A1 expression specifically when activating STAT1. This suggests that CXCL10 exacerbates septic liver injury mainly by activating the STAT1/SLC11A1 axis to promote NETs formation. Therefore, CXCL10 may serve as a potential therapeutic target for septic liver injury, and neutralizing antibodies against CXCL10 hold promise for alleviating liver damage and even reversing liver failure.

## 5 Conclusion

In conclusion, this study reveals a liver‑specific mechanism driving neutrophil‑mediated liver injury during sepsis. We identified that hepatocyte‑secreted CXCL10 recruits neutrophils and upregulates the metal transporter SLC11A1 within these cells. SLC11A1 subsequently promotes Fe^2^^+^‑dependent ROS burst and NETs formation, which in turn polarizes macrophages toward a pro‑inflammatory phenotype, thereby amplifying hepatic damage. Clinically, circulating CXCL10 levels correlate with liver injury severity in septic patients. Importantly, CXCL10 neutralization or knockdown reduces NETs formation and attenuates liver injury in septic mice. Collectively, these findings establish the hepatocyte‑originated “CXCL10–SLC11A1–NETs” axis as a key pathogenic pathway and a promising therapeutic target for sepsis‑induced liver injury.

## Author Contributions

**Shian Yu**, **Min Yu**, and **Lude Wang**: validation, supervision, software, resources, project administration, methodology, investigation, funding acquisition, formal analysis, data curation. **Haiping Lin**: writing – review & editing, writing – original draft, visualization, validation, supervision, software, resources, project administration, methodology, investigation, funding acquisition, formal analysis, data curation, conceptualization. **Shicong Zheng** and **Pei Song**: methodology. **Jie Chang**, **Zewei Chen**, and **Cang Li**: writing – review & editing, writing – original draft, visualization, validation.

## Funding

This work was supported by the China Postdoctoral Science Foundation (Grant No. 2025M782270), the Zhejiang Provincial Medical and Health Science and Technology Project (Grant Nos. 2023KY381 and 2025HY1354), the Jinhua Science and Technology Program (Grant Nos. 2023‐3‐066 and 2026‐3‐056), and the Jinhua Central Hospital Postdoctoral Research Startup Fund (Grant No. 2024‐9‐002).

## Ethical Statement

The animal experiments were approved by the Experimental Animal Welfare and Ethics Committee of Jinhua Hospital, affiliated with Zhejiang University School of Medicine (AL‐JHYY2025199). The clinical study was conducted in accordance with the World Medical Association's Declaration of Helsinki (2013) and the Declaration of Istanbul (2018). Biological specimens were obtained from Jinhua Hospital, affiliated with Zhejiang University School of Medicine. Written informed consent was obtained from all participants, and the study protocol was formally approved by the institutional Ethics Committee (2021‐Ethics Review‐319).

## Conflicts of Interest

The authors declare no conflicts of interest.

## Supporting information




**Supporting File 1**: advs76424‐sup‐0001‐SuppMat.docx.

**Supporting File 2**: advs76424‐sup‐0002‐TableS1.docx.

**Supporting File 3**: advs76424‐sup‐0003‐TableS2.docx.

## Data Availability

The raw sequence data reported in this paper have been deposited in the Genome Sequence Archive (Genomics, Proteomics & Bioinformatics 2025) in National Genomics Data Center (Nucleic Acids Res 2025), China National Center for Bioinformation / Beijing Institute of Genomics, Chinese Academy of Sciences (GSA: CRA042217) that are publicly accessible at https://ngdc.cncb.ac.cn/gsa.
