# Supplementary material for: CXCL10/SLC11A1 Axis Exacerbates Septic Liver Injury by Regulating Neutrophil Extracellular Traps Formation to Drive Macrophage Pro‑Inflammatory Polarization
Source: Adv Sci (Weinh). 2026 Jul 13;13(54):e76424. doi: 10.1002/advs.76424 (PMC13360119; doi:10.1002/advs.76424)
Supplement: Supplementary file 3 [file ADVS-13-e76424-s003.docx]

| **Supplementary Table 1. Oligonucleotides，recombinant DNA and software used in the study** | | | |
| --- | --- | --- | --- |
| **Reagent or Resource** | **Source** | **Identifier** |  |
| **Oligonucleotides** | | | |
| Rela shRNA_1 | TCCGGAATCTAAGACCATCAA | N/A |  |
| Rela shRNA_2 | GCCAGTGAGAATGAGGGCCAT | N/A |  |
| Rela shRNA_3 | GATGTCTGAATCCGGAATCTA | N/A |  |
| RT-qPCR primer-*Slc11a1* | ACTGCTTATCTGGCCTGGAC CTCTTTGCTTGCTGGATGCC | N/A |  |
| RT-qPCR primer-*Cxcl10* | TCTGAGTGGGACTCAAGGGAT AGGCTCGCAGGGATGATTTC | N/A |  |
| RT-qPCR primer-*ACTIN* | TATAAAACCCGGCGGCGCA TCATCCATGGCGAACTGGTG | N/A |  |
| ChIP-qPCR primer-*Cxcl10* | TTCTGCAAGGCACTGCATCT CCCTGTAAACCGAGGGCATT | N/A |  |
| **Recombinant DNA** | | | |
| pRL-TK | This paper | N/A |  |
| pGL3-Basic-Luc | This paper | N/A |  |
| pGL3-*Cxcl10* WT-Luc | This paper | N/A |  |
| pGL3-*Cxcl10* (-666—+100)-Luc | This paper | N/A |  |
| pGL3-*Cxcl10* (-1334—-667; +1—+100)-Luc | This paper | N/A |  |
| pGL3-*Cxcl10* (-2000—-1335; +1—+100)-Luc | This paper | N/A |  |
| pGL3- *Cxcl10* MBS1 Mut-Luc | This paper | N/A |  |
| pGL3- *Cxcl10* MBS2 Mut-Luc | This paper | N/A |  |
| pGL3- *Cxcl10* MBS3 Mut-Luc | This paper | N/A |  |
| pGL3- *Cxcl10* MBS4 Mut-Luc | This paper | N/A |  |
| **Software and algorithms** | | | |
| SPSS 22.0 | IBM | https://www.ibm.com/cn-zh/spss |  |
| R 4.4.2 | N/A | https://cran.r-project.org/ |  |
| Jupyter 6.4.0 | N/A | https://github.com/jupyter/notebook |  |
| Prism 9.0 | GraphPad | https://www.graphpad.com/ |  |
| ImageJ | N/A | https://imagej.nih.gov/ij/ |  |
| FlowJo 10.8 | N/A | https://www.flowjo.com/ |  |
