# Supplementary material for: CXCL10/SLC11A1 Axis Exacerbates Septic Liver Injury by Regulating Neutrophil Extracellular Traps Formation to Drive Macrophage Pro‑Inflammatory Polarization
Source: Adv Sci (Weinh). 2026 Jul 13:e76424. Online ahead of print. doi: 10.1002/advs.76424 (PMC13360119; doi:10.1002/advs.76424)
Supplement: Supplementary file 1 — Supporting File 1: advs76424‐sup‐0001‐SuppMat.docx. [file ADVS-9999-e76424-s001.docx]

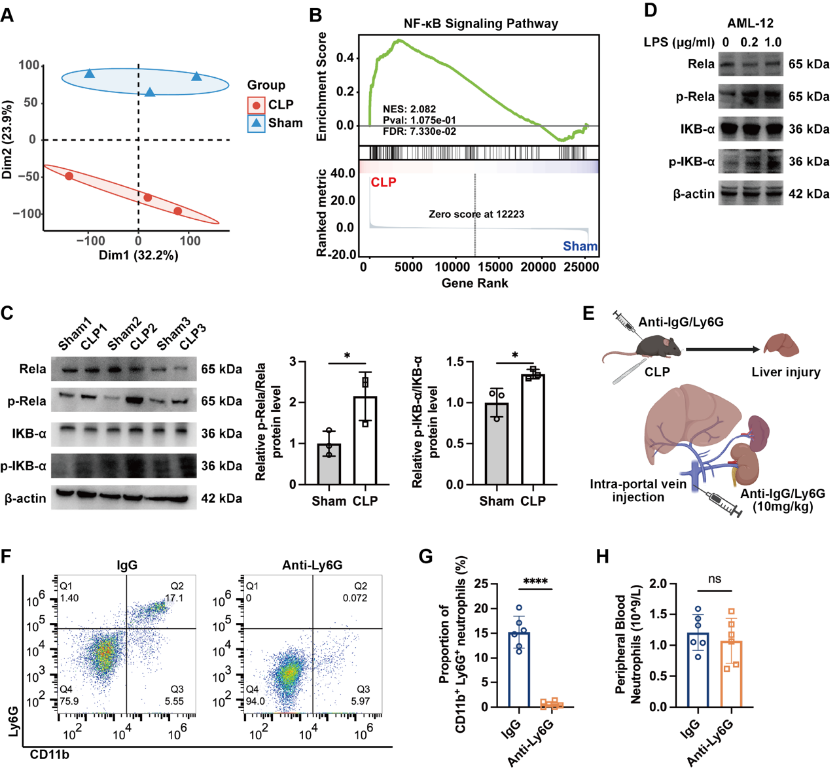


**Supplementary Figure 1.** (A) Principal component analysis (PCA) of RNA‑seq data derived from liver tissues of the CLP and Sham groups. (B) Enrichment plot for the NF-κB signaling pathway gene set in CLP versus Sham comparison. The normalized enrichment score (NES) and false‑discovery rate (FDR) are indicated. (C) Immunoblot analysis of NF‑κB signaling pathway proteins (RelA, phospho‑RelA, IκB‑α, phospho‑IκB‑α) in liver tissues from Sham and CLP groups. (D) Immunoblot analysis of NF‑κB signaling pathway proteins in AML‑12 cells treated with 0, 0.2, and 1 μg/mL LPS for 24 hours. (E) Schematic illustration of intra-portal vein injection of either isotype control antibody or anti‑Ly6G in mice. (F, G) Flow cytometric analysis (F) and quantification (G) of the proportion of neutrophils (identified as CD11b⁺ Ly6G⁺ cells) among CD45⁺ cells in the livers of CLP mice treated with either isotype control antibody or anti‑Ly6G (10 mg/kg), assessed 24 hours post‑surgery. (H) The proportion of neutrophils in the peripheral blood of CLP mice treated with either isotype control antibody or anti‑Ly6G (10 mg/kg) , assessed 24 hours post‑surgery. In (C), (G), (H) data represent mean ± SD; unpaired two-tailed Student’s t test. *P < 0.05, ****P < 0.0001 and ns P > 0.05 between the indicated groups.


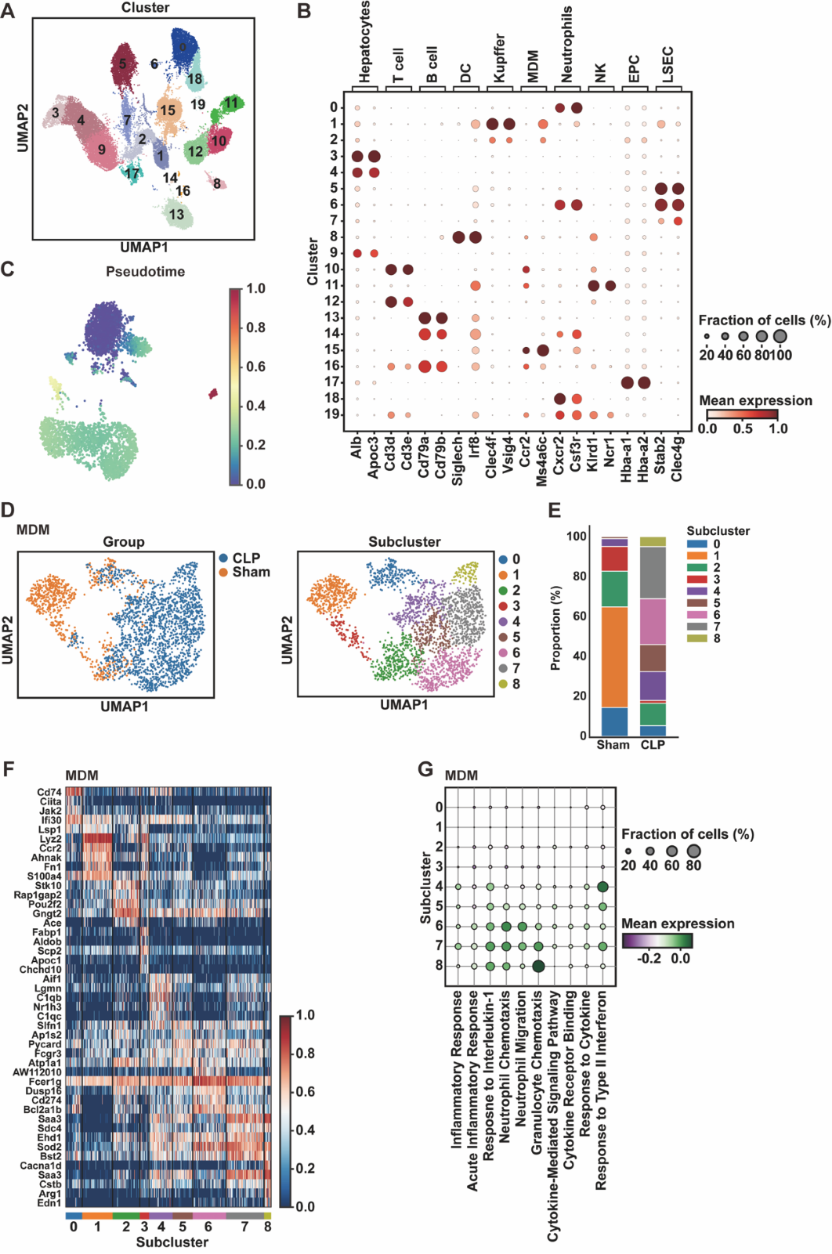


**Supplementary Figure 2.** (A) UMAP visualization of scRNA‑seq data from liver‑derived cells, colored according to unsupervised Leiden clustering. (B) Dot plot of canonical marker genes used for cell type annotation. Dot size represents the percentage of cells expressing the gene within each cluster; color intensity represents the average expression level. (C) UMAP showing the scRNA-seq differentiation trajectory of immature neutrophils towards mature neutrophils. Cells are colored by pseudotime. (D) UMAP of MDMs from the CLP and Sham groups. MDMs were divided into 9 distinct cell subclusters through unsupervised clustering. (E) The proportions of distinct MDM subclusters in the CLP and Sham groups. (F) Heatmap showing the highly expressed genes within each of the 9 distinct MDM subclusters. (G) Dot plot of GSVA scores showing the enrichment of significant GO pathways (top 10 ranked by adjusted p‑value) across different MDM subclusters.


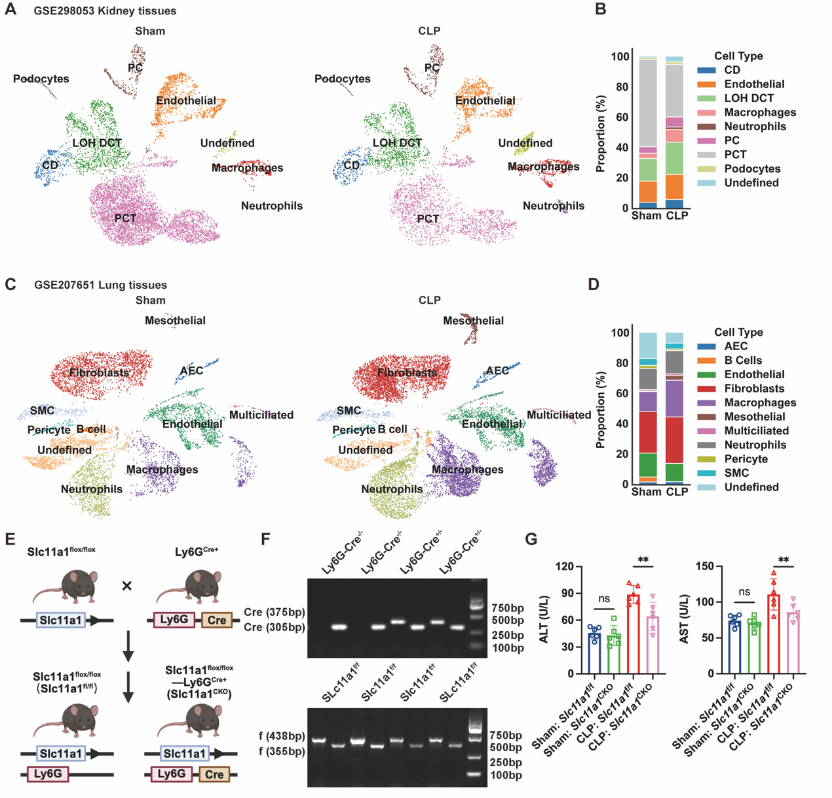


**Supplementary Figure 3.** (A) UMAP visualization of kidney scRNA-seq data (GSE298053). Cells were annotated based on canonical marker genes as follows: collecting duct cells (CD), endothelial cells, distal convoluted tubule cells of the loop of Henle (LOH DCT), macrophages, neutrophils, principal cells (PC), proximal convoluted tubule cells (PCT), and podocytes. (B) The proportions of distinct cell types in kidney scRNA-seq data from the CLP and Sham groups. (C) UMAP visualization of lung scRNA-seq data (GSE207651). Cells were annotated based on marker expression as: alveolar epithelial cells (AEC), B cells, endothelial cells, fibroblasts, macrophages, mesothelial cells, multiciliated cells, neutrophils, pericytes, and smooth muscle cells (SMC). (D) The proportions of distinct cell types in lung scRNA-seq data from the CLP and Sham groups. (E) Schematic illustration of the breeding strategy used to generate neutrophil‑specific *Slc11a1* conditional knockout mice (*Ly6G*-Cre^+^ *Slc11a1*^f/f^, referred to as *Slc11a1*^CKO^) and control mice (*Ly6G*-Cre^-^ *Slc11a1*^f/f^, referred to as *Slc11a1*^f/f^). (F) Representative agarose gel electrophoresis results of genotyping PCR for *Slc11a1*^CKO^ and *Slc11a1*^f/f^ mice. (G) Quantification of serum ALT and AST levels in *Slc11a1*^CKO^ mice compared with *Slc11a1*^f/f^ mice in the Sham and CLP model, assessed 24 hours post‑surgery. In (G) data represent mean ± SD; one-way ANOVA with multiple comparisons test. **P < 0.01 and ns P > 0.05 between the indicated groups.


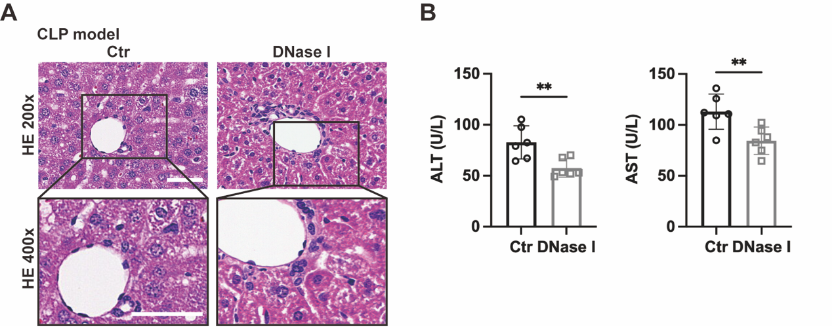


**Supplementary Figure 4.** (A) Representative H&E staining images of liver injury in CLP mice (*Slc11a1*^f/f^ vs. *Slc11a1*^CKO^), assessed 24 hours post‑surgery. Scale bar = 50 µm. (B) Quantification of serum ALT and AST levels in the CLP model (*Slc11a1*^f/f^ vs. *Slc11a1*^CKO^), assessed 24 hours post‑surgery. In (B) data represent mean ± SD; unpaired two-tailed Student’s t test. **P < 0.01 between the indicated groups.


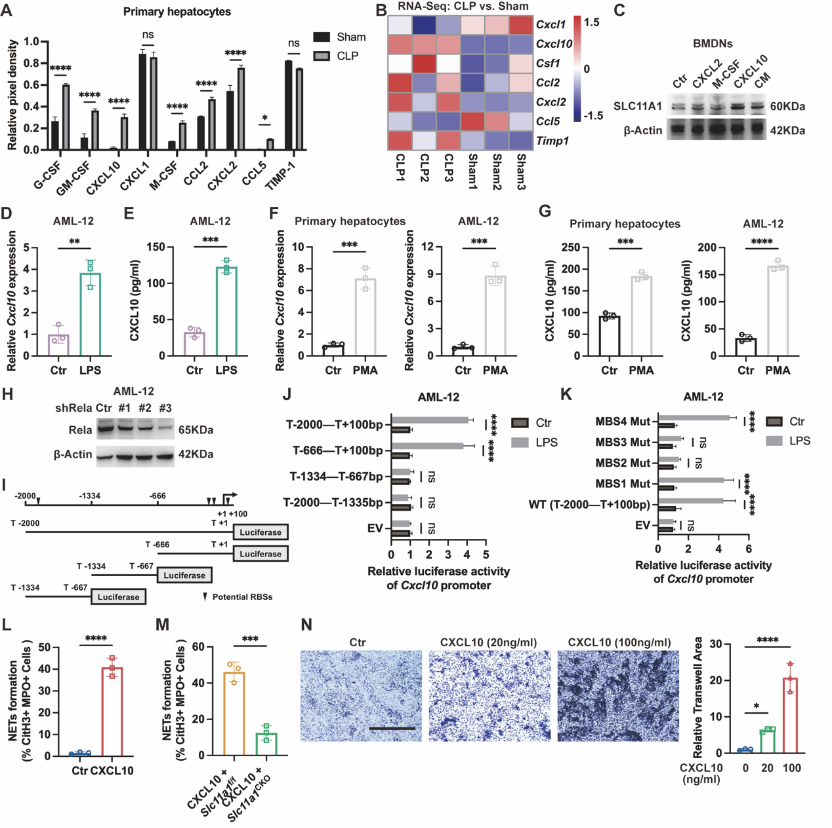


**Supplementary Figure 5.** (A) Quantitative analysis of cytokine secretion levels in the cytokine array performed on primary hepatocytes isolated from the livers of Sham or CLP groups. (B) Heatmap of RNA-Seq data showing normalized expression levels of selected cytokine and chemokine genes in liver tissues of CLP versus Sham groups. Expression values are row‑scaled (z‑score normalized) across samples. (C) Immunoblot analysis of SLC11A1 protein expression in BMDNs treated with recombinant CXCL2, CXCL10, M-CSF (100 ng/ml each), conditioned medium (CM) from primary hepatocytes isolated from CLP model mice, or vehicle control for 24 hours. (D, E) *Cxcl10* mRNA levels (D) and CXCL10 secretion levels (E) in AML-12 treated with LPS (1 μg/ml) or vehicle control for 24 hours, determined by RT-qPCR and ELISA, respectively. (F) *Cxcl10* mRNA levels in primary hepatocytes or AML-12 treated with PMA (100 ng/ml) or vehicle control for 24 hours, determined by RT-qPCR. (G) CXCL10 secretion levels in primary hepatocytes or AML-12 treated with PMA (100 ng/ml) or vehicle control for 24 hours, determined by ELISA. (H) Immunoblot analysis of Rela protein expression in AML‑12 cells with stable Rela knockdown or control (scrambled shRNA). (I) Schematic illustration of the *Cxcl10* promoter-luciferase reporter constructs used for mapping Rel-responsive elements. (J) Relative luciferase activity of the *Cxcl10* promoter in AML‑12 cells transfected with different truncated *Cxcl10* promoter constructs and treated with or without LPS (1 μg/ml) for 24 hours. (K) Relative luciferase activity of the *Cxcl10* promoter in AML‑12 cells transfected with different *Cxcl10* promoter mutants and treated with or without LPS (1 μg/ml) for 24 hours. (L) Quantification of NETs formation in BMDNs treated with or without recombinant CXCL10 (100 ng/ml) for 24 hours, defined as % MPO⁺/Cit H3⁺ cells with >2-fold mean nuclear size. (M) Quantification of NETs formation in BMDNs from *Slc11a1*^f/f^ and *Slc11a1*^CKO^ mice treated with recombinant CXCL10 (100 ng/ml) for 24 hours, defined as % MPO⁺/Cit H3⁺ cells with >2-fold mean nuclear size. (N) Neutrophil chemotaxis assay of BMDNs treated with recombinant CXCL10 (0, 20, 100 ng/mL) for 4 hours. The bar graph quantifies the relative migration area (normalized to 0 ng/mL control). Scale bar = 100 µm. In (A), (D), (E), (F), (G), (J), (K), (L), (M) data represent mean ± SD; unpaired two-tailed Student’s t test. In (N) data represent mean ± SD; one-way ANOVA with multiple comparisons test. *P < 0.05, **P < 0.01, ***P < 0.001, ****P < 0.0001 and ns P > 0.05 between the indicated groups.


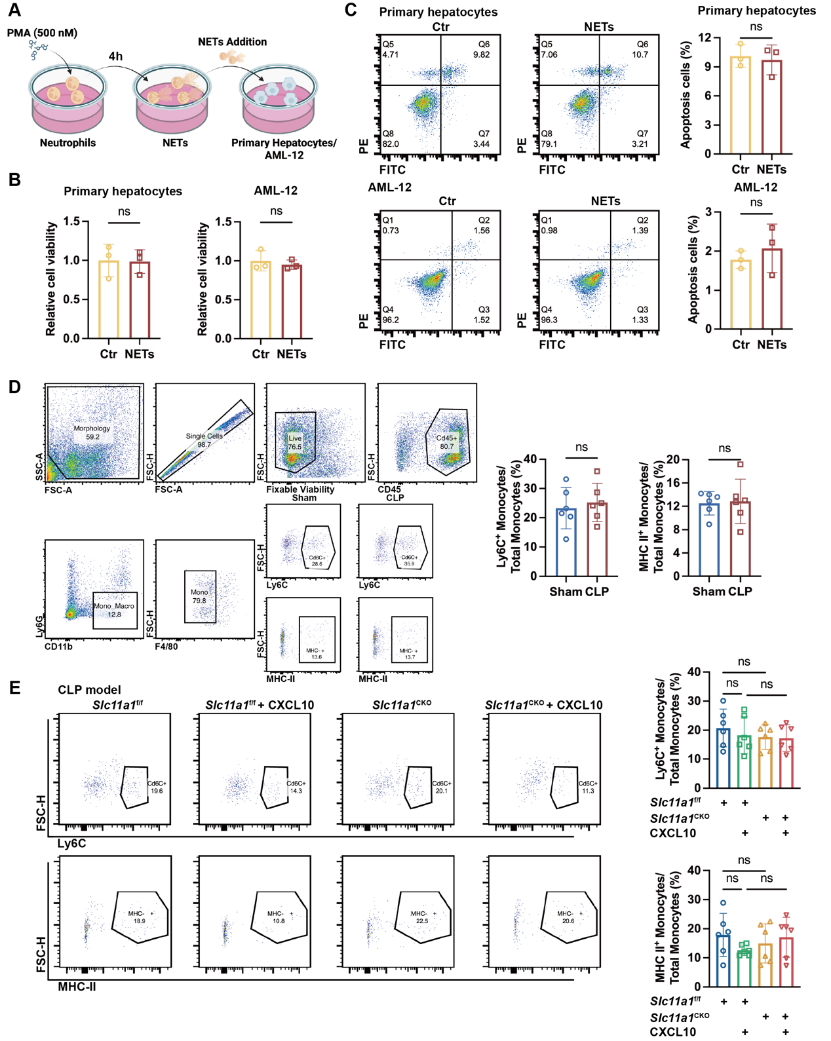


**Supplementary Figure 6.** (A) Schematic illustration of the experimental procedure: Neutrophils were stimulated with 500 nM PMA for 4 hours to induce NETs formation. The generated NETs were then isolated via high-speed centrifugation and subsequently added to the culture medium of primary hepatocytes or AML-12 cells (2 μg/ml). (B) The cell viability of primary hepatocytes and AML-12 cells treated with or without NETs (2 μg/ml) for 24 hours. (C) Apoptosis of primary hepatocytes and AML-12 cells treated with or without NETs (2 μg/ml) for 24 hours, assessed by flow cytometry. Cells positive for both Annexin V and PI were defined as apoptotic cells. (D) Flow cytometric analysis of Ly6C^+^ and MHC II^+^ monocyte proportions in the Sham and CLP groups 24 hours post‑surgery, with the gating strategy shown. (E) Flow cytometric analysis of CD86^+^ and CD206^+^ monocyte proportions in *Slc11a1*^CKO^ and *Slc11a1*^f/f^ mice, with or without CXCL10 (100 ng/ml) treatment, following CLP, assessed 24 hours post‑surgery. In (B), (C), (D) data represent mean ± SD; unpaired two-tailed Student’s t test. In (E) data represent mean ± SD; one-way ANOVA with multiple comparisons test. *P < 0.05, **P < 0.01, ***P < 0.001 and ns P > 0.05 between the indicated groups.


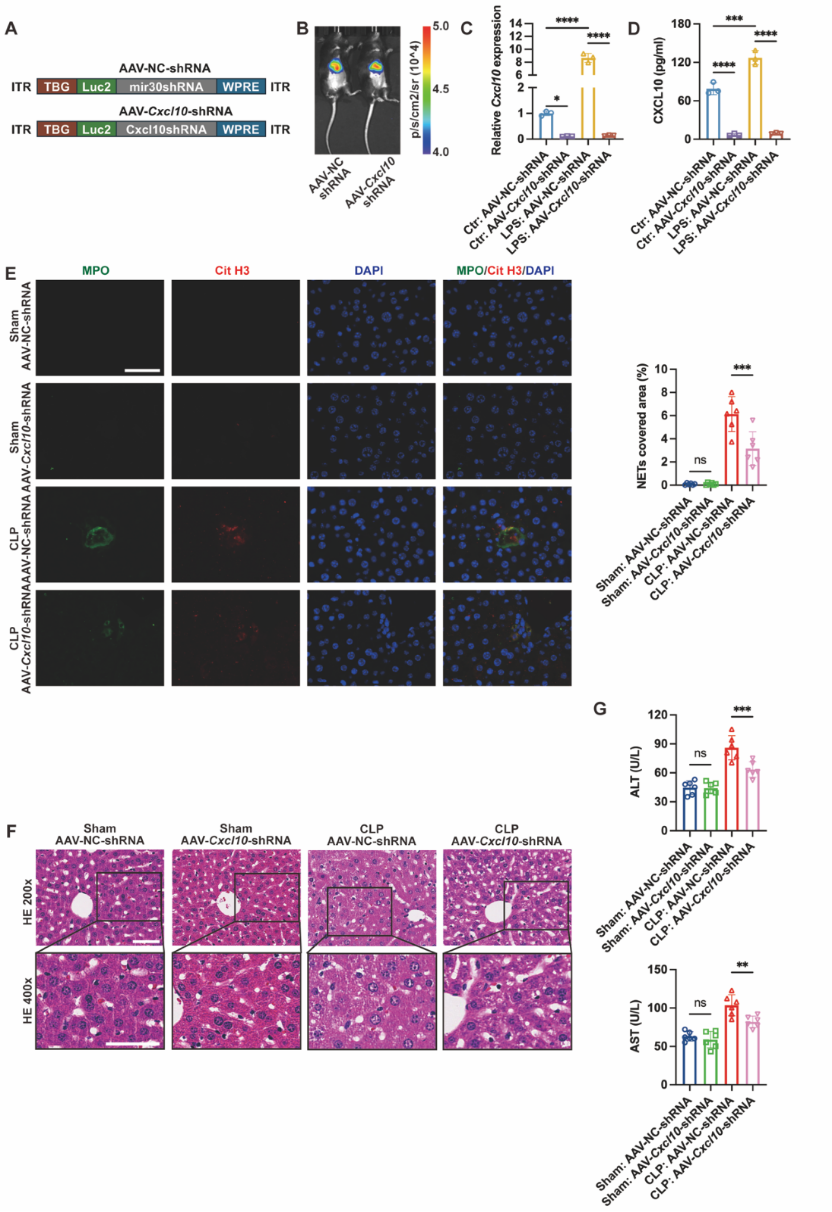


**Supplementary Figure 7.** (A) Schematic illustration of the construction of AAV‑NC‑shRNA and AAV‑*Cxcl10*‑shRNA. (B) Four weeks following tail vein injection of AAV‑NC‑shRNA or AAV‑*Cxcl10*‑shRNA into mice, *in vivo* bioluminescence imaging (IVIS) was performed to assess the fluorescence distribution. (C, D) *Cxcl10* mRNA levels and CXCL10 secretion levels in primary hepatocytes isolated from mice treated with AAV‑NC‑shRNA or AAV‑Cxcl10‑shRNA, with or without LPS (1 μg /mL) stimulation for 24 hours, determined by RT‑qPCR and ELISA, respectively. (E) NETs formation assessed in liver tissues of mice subjected to Sham or CLP surgery and treated with either AAV‑NC‑shRNA or AAV‑Cxcl10‑shRNA, visualized by immunofluorescence co‑staining of MPO and Cit‑H3, assessed 24 hours post‑surgery. (F) Representative H&E staining images of liver injury in mice subjected to Sham or CLP surgery and treated with either AAV‑NC‑shRNA or AAV‑*Cxcl10*‑shRNA, assessed 24 hours post‑surgery. (G) Quantification of serum ALT and AST levels in mice subjected to Sham or CLP surgery and treated with either AAV‑NC‑shRNA or AAV‑*Cxcl10*‑shRNA, assessed 24 hours post‑surgery. In (C), (D), (E), (G) data represent mean ± SD; one-way ANOVA with multiple comparisons test. *P < 0.05, **P < 0.01, ***P < 0.001, ****P < 0.0001 and ns P > 0.05 between the indicated groups.
