# Supplementary material for: CXCL10/SLC11A1 Axis Exacerbates Septic Liver Injury by Regulating Neutrophil Extracellular Traps Formation to Drive Macrophage Pro‑Inflammatory Polarization
Source: Adv Sci (Weinh). 2026 Jul 13:e76424. Online ahead of print. doi: 10.1002/advs.76424 (PMC13360119; doi:10.1002/advs.76424)
Supplement: Supplementary file 3 — Supporting File 3: advs76424‐sup‐0003‐TableS2.docx. [file ADVS-9999-e76424-s003.docx]

| **Supplementary Table 2. Reagent and resources used in the study** | | |
| --- | --- | --- |
| **Reagent or Resource** | **Source** | **Identifier** |
| **Antibodies** | | |
| CD11b antibody | BD Pharmingen | #553310 |
| Ly6G antibody (for FC) | BD Pharmingen | #551461 |
| F4/80 antibody | BD Pharmingen | #565410 |
| CD86 antibody | BD Pharmingen | #757581 |
| CD206 antibody | BD Pharmingen | #568808 |
| CD45 antibody | BD Pharmingen | #567111 |
| Ly6C antibody | BD Pharmingen | #560525 |
| MHC II antibody | BD Pharmingen | #743870 |
| NF-kappaB p65 antibody | Cell Signaling Technology | #8242 |
| Phospho-NF-kappaB p65 (Ser536) antibody | Cell Signaling Technology | #3033 |
| IkappaB alpha antibody | Cell Signaling Technology | #4812 |
| Phospho-IkappaB alpha (Ser32) antibody | Cell Signaling Technology | #2859 |
| Ly6G antibody (for IHC) | Cell Signaling Technology | #87048 |
| Slc11a1 antibody | Thermo Fisher Scientific | #PA5-114373 |
| Anti-Histone H3 (citrulline R2 + R8 + R17) antibody | Abcam | #ab281584 |
| MPO antibody | MedChemExpress | #HY-P85882 |
| Stat1 antibody | Cell Signaling Technology | #9172 |
| Phospho-Stat1 antibody | Cell Signaling Technology | #9167 |
| Stat3 antibody | Cell Signaling Technology | #9139 |
| Phospho-Stat3 antibody | Cell Signaling Technology | #9145 |
| CD86 | Proteintech | #30691-1-AP |
| CD206 | Proteintech | #83485-1-RR |
| InVivoMAb anti-mouse Ly6G | Bio X Cell | #1A8 |
| InVivoMAb anti-mouse CXCL10 | Bio X Cell | #1F11 |
| Mouse FcR Blocking Reagent (Anti mouse CD16/CD32) | Starter | #S0B0599 |
| β-Actin antibody | Beyotime | #AF5003 |
| **Bacterial and virus strains** | | |
| Competent E.coli DH5a | WEIDI Biosciences | #DL1001M |
| **Biological samples** | | |
| Peripheral blood from septic patients | Jinhua Municipal Central Hospital | N/A |
| **Critical commercial assays** | | |
| LPS | MedChemExpress | #HY-D1056 |
| PMA | MedChemExpress | #HY-18739 |
| Fludarabine | MedChemExpress | #HY-B0069 |
| STAT3-IN-1 | MedChemExpress | #HY-100753 |
| Alanine aminotransferase assay kit | Purui Biotechnology | #ALT01 |
| Aspartate aminotransferase assay kit | Purui Biotechnology | #AST01 |
| Zombie NIR™ Fixable Viability Kit | Biolegend | #423106 |
| Protease inhibitor cocktail | Selleck | #B14001 |
| Phosphatase inhibitor cocktail | Selleck | #B15001 |
| DAPI | Beyotime | #C1341S |
| Lipofectamine 3000 | Thermo Fisher Scientific | #L3000015 |
| Puromycin | Yeasen | #60210ES25 |
| Collagenase IV | Sigma | #C5138 |
|  | Sigma | #10104159001 |
| BD Cytofix/Cytoperm™ Fixation/Permeabilization Kit | BD Pharmingen | #554714 |
| Mouse FcR Blocking Reagent | STARTER | #S0B0599 |
| MojoSort™ Mouse Neutrophil Isolation Kit | BioLegend | #480058 |
| mCytokine Array Kit | R&D Systems | #ARY006 |
| Dual Luciferase Reporter Assay Kit | Vazyme | #DL101-01 |
| SimpleChIP® Plus Enzymatic Chromatin IP Kit (Magnetic Beads) | Cell Signaling Technology | #9005 |
| Mouse CXCL-10/IP-10 ELISA Kit | Multi Sciences | #EK268 |
| Human CXCL-10/IP-10 ELISA Kit | Multi Sciences | #EK168 |
| Zombie NIR™ Fixable Viability Kit | Biolegend | #423106 |
| CCK-8 Cell Counting Kit | Vazyme | #A311-01 |
| cDNA Synthesis Kit | YEASEN | #11121ES60 |
| SYBR Green Master Mix | YEASEN | #11202ES60 |
| FerroOrange probe | MedChemExpress | # HY-D1913 |
| DCFDA/H₂DCFDA probe | Abcam | #ab113851 |
| 6.5 mm Transwell® with 5.0 µm Pore Polycarbonate Membrane Insert, Sterile | Corning | #3421 |
| 6.5 mm Transwell® with 3.0 µm Pore Polycarbonate Membrane Insert, Sterile | Corning | #3420 |
| **Experimental models** | | |
| C57BL/6J mice | GemPharmatech | #N000013 |
| C57BL/6J-Slc11a1^em1Cflox^ mice | GemPharmatech | #T018997 |
| Ly6G-iCre mice | Cyagen | #C001310 |
